# Optimizing cognitive neuroscience experiments for separating event- related fMRI BOLD responses in non-randomized alternating designs

**DOI:** 10.3389/fnimg.2023.1068616

**Published:** 2023-04-17

**Authors:** Soukhin Das, Weigang Yi, Mingzhou Ding, George R. Mangun

**Affiliations:** ^1^Center for Mind and Brain, University of California, Davis, Davis, CA, United States; ^2^Department of Psychology, University of California, Davis, Davis, CA, United States; ^3^Pruitt Family Department of Biomedical Engineering, University of Florida, Gainesville, FL, United States; ^4^Department of Neurology, University of California, Davis, Davis, CA, United States

**Keywords:** fMRI, deconvolution, BOLD, optimization, cognitive, neuroscience, experiment, design

## Abstract

Functional magnetic resonance imaging (fMRI) has revolutionized human brain research. But there exists a fundamental mismatch between the rapid time course of neural events and the sluggish nature of the fMRI blood oxygen level-dependent (BOLD) signal, which presents special challenges for cognitive neuroscience research. This limitation in the temporal resolution of fMRI puts constraints on the information about brain function that can be obtained with fMRI and also presents methodological challenges. Most notably, when using fMRI to measure neural events occurring closely in time, the BOLD signals may temporally overlap one another. This overlap problem may be exacerbated in complex experimental paradigms (stimuli and tasks) that are designed to manipulate and isolate specific cognitive-neural processes involved in perception, cognition, and action. Optimization strategies to deconvolve overlapping BOLD signals have proven effective in providing separate estimates of BOLD signals from temporally overlapping brain activity, but there remains reduced efficacy of such approaches in many cases. For example, when stimulus events necessarily follow a non-random order, like in trial-by-trial cued attention or working memory paradigms. Our goal is to provide guidance to improve the efficiency with which the underlying responses evoked by one event type can be detected, estimated, and distinguished from other events in designs common in cognitive neuroscience research. We pursue this goal using simulations that model the nonlinear and transient properties of fMRI signals, and which use more realistic models of noise. Our simulations manipulated: (i) Inter-Stimulus-Interval (ISI), (ii) proportion of so-called null events, and (iii) nonlinearities in the BOLD signal due to both cognitive and design parameters. We offer a theoretical framework along with a python toolbox called deconvolve to provide guidance on the optimal design parameters that will be of particular utility when using non-random, alternating event sequences in experimental designs. In addition, though, we also highlight the challenges and limitations in simultaneously optimizing both detection and estimation efficiency of BOLD signals in these common, but complex, cognitive neuroscience designs.

## 1. Introduction

Functional magnetic resonance imaging (fMRI) is a powerful method for understanding the functional anatomy of the human brain (e.g., Kwong et al., 1992; Ogawa et al., 1992; Glover, 2011). In cognitive neuroscience, fMRI has provided a rich view on the organization of human perception and cognition (e.g., Corbetta et al., 2008; D'Esposito and Badre, 2012). Event-related fMRI is highly effective for analyzing data from common cognitive-experimental designs (e.g., McCarthy et al., 1997; Buckner, 1998; Huettel, 2012; Liu, 2012). For example, such approaches are regularly applied to answer questions about perception, cognition, and action (Kastner et al., 1999; Corbetta et al., 2000; Jha and McCarthy, 2000; Hopfinger et al., 2001; Winterer et al., 2002; Ranganath et al., 2003).

One challenge for event-related methods in cognitive neuroscience is the sluggish and delayed nature of the brain's hemodynamic response. The hemodynamic response unfolds over the course of seconds, whereas the underlying associated neural processes take place with millisecond timing. Therefore, the events of interest in the brain in many experimental designs may occur more closely together in time than can be easily resolved from the blood oxygenation-level dependent (BOLD) signals we acquire with fMRI. These basic facts present challenges, the first being to measure brain responses to the events of interest separately from those related to other temporally and spatially overlapping events (e.g., one sensory signal vs. another) (e.g., Burock et al., 1998).

Optimization strategies to deconvolve overlapping BOLD signals have proven effective in providing separate estimates of BOLD signals from temporally overlapping brain activity. A number of studies have shown highly reliable event-related fMRI estimates using randomized event sequences with second and even sub-second interstimulus intervals (ISIs) (Buckner, 1998; Burock et al., 1998; D'Esposito et al., 1999; Hinrichs et al., 2000). Josephs and Henson (1999) characterized the relative fitness and efficiency of random event sequences. They generated random event sequences that encompass the space of varying parameters of ISIs of stochastic and jittered variations in event onset times (Burock et al., 1998; Friston et al., 1999). Dale (1999) suggested jittering the time interval between onsets of consecutive stimuli, recommending that the average of these intervals should be kept small. Later, this deconvolution approach was further generalized by Friston et al. (1999) for different combinations of conditions, increasing the efficiency of fMRI analyses. This was an important advancement because most cognitive neuroscientists are interested in multiple contrasts in a single study, for instance, the difference in activation between different conditions and the baseline, or the difference between a treatment and a control condition (Huettel, 2012; Liu, 2012).

**Figure 1:**
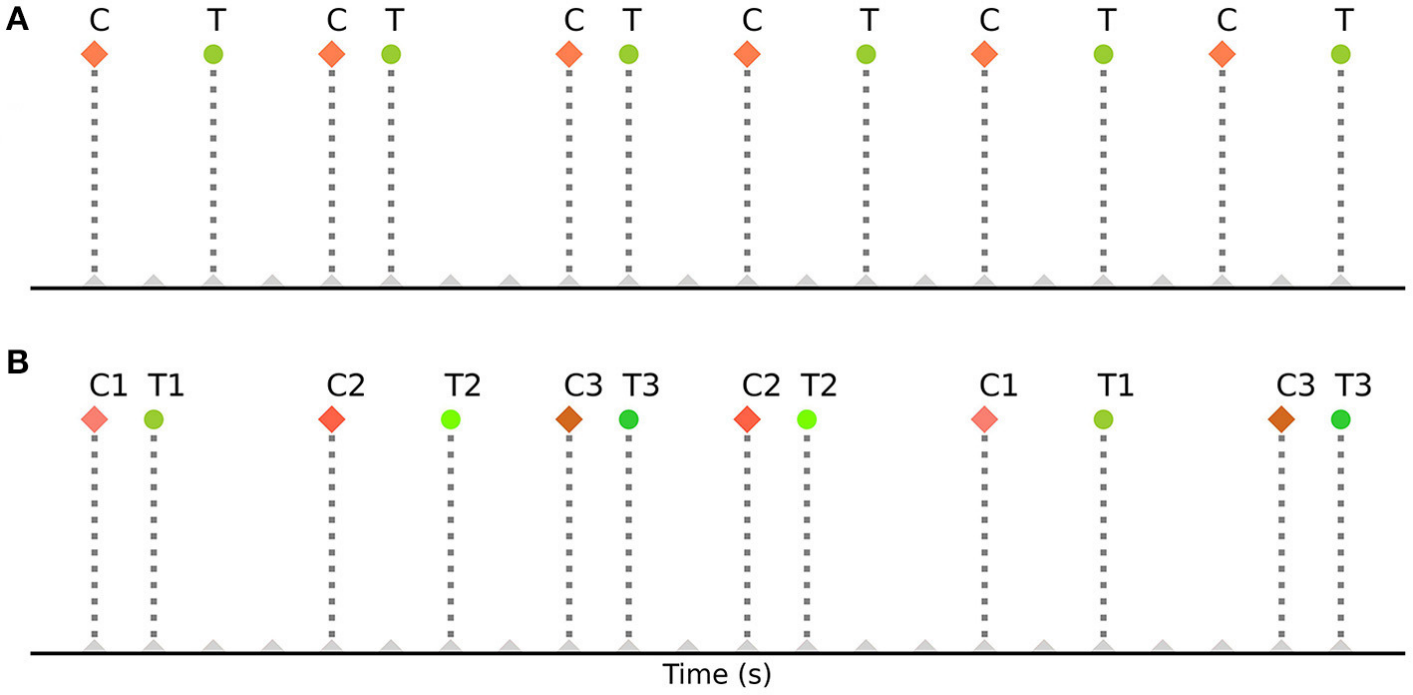
Schematic representation of the entire simulation framework. Divided into two distinct pipelines, the **(A)** portion of the figure represents the noise pipeline, and the **(B)** shows the response signal pipeline. Finally, they are added together to obtain the final simulated signal for a specific set of parameters.

These strategies have also helped in determining the most efficient sequence and timing of events for experimental designs. However, they are based on strategies such as randomization of events, orthogonal design of the design matrix (Liu et al., 2001), deterministic jitter of their onset timings, and specialized sequencing (m- sequences) (Buračas and Boynton, 2002; Kao et al., 2009), which may be difficult or impossible to implement in some common cognitive neuroscience experiments. For example, in some designs the order of events cannot be fully randomized, such as in cue-target attention paradigms where the events (cue stimuli and target stimuli) repeat in an alternating fashion (Hopfinger et al., 2000b; Taylor et al., 2008; Rajan et al., 2019). In such alternating event-related designs, the sequence of events is fixed and predetermined. These paradigms typically involve presenting a cue to direct a participant's attention, followed by a target stimulus that requires a response. The order of events (cues and targets) remains fixed throughout the design. During each case, a cue is always followed by a corresponding target. Figure 1A illustrates a basic cue-target paradigm with a single cue (C) - target (T) pair repeating on a trial-by-trial basis (CTCTCT…), while Figure 1B demonstrates its extension to multiple cue-target pairs (example codes can be found in the toolbox repository: https://github.com/soukhind2/deconv).

Over the years, some studies have attempted to address the analytic challenges in these situations where events alternate and cannot, therefore, be completely randomized. Some have examined various fitness criteria for alternating event-related designs (Josephs and Henson, 1999; Liu and Frank, 2004; Ruge et al., 2009), while others have tried to explore different parameters to minimize the overlap (Huettel et al., 2004; Lütkenhöner, 2010; Liu, 2012). Recently, Prince et al. (2022) developed GLMsingle, a data-driven single-trial approach to deconvolve events close together in time. It uses techniques such as appropriate hemodynamic response function (HRF) fitting, data-driven denoising of signals, and regularization of weights in fMRI regression models to estimate single-trial responses and optimize detection efficiency (see also, Turner et al., 2012; Abdulrahman and Henson, 2016). Nevertheless, as it can only be employed after fMRI data collection, it is insufficient to determine the best parameters during the initiation and design phase of any experiment (though it can be used with already available fMRI datasets to get some inference). Therefore, despite such important efforts, there has been no direct and detailed quantification of the parameters used to assemble a sequence of constrained and repetitive events for a design (for example, bounds of inter-stimulus-interval, proportion of null trials, contextual factors). Despite these important efforts, there has been no detailed quantification of the parameters used to assemble a sequence of constrained and repetitive events for a design (for example, bounds of inter-stimulus-interval, proportion of null trials, and contextual factors). Furthermore, it is unclear how changing specific design parameters affects their efficacy in creating efficient event-related designs. We aim to bridge this gap by exploring a large range of combinations of design parameters and strategies that are appropriate for alternating event-related designs in cognitive neuroscience research. Finally, we introduce, *deconvolve*, a Python-based toolbox to facilitate the implementation of the theory and techniques used in this work.

Using simulations, our approach involves a comprehensive search for estimation efficiency and detection power over the space defined by the bounds of typical design parameters. We implemented a realistic model of nonlinearity for our simulations. We also included a more realistic noise component in our simulations by using the excellent tools provided in fmrisim, a python package developed by Ellis et al. (2020), which extracts statistically accurate noise properties from fMRI data. In addition, we attempt to consider the nonlinear properties of BOLD signals that are introduced in many event-related experimental designs. We will describe a “fitness landscape”, whose dimensions are governed by the different parameters of interest. This landscape will serve as a reference for creating optimal experimental designs for many common cognitive neuroscience research questions.

## 2. Methods

### 2.1. Modeling alternating event-related responses

**Figure 2:**
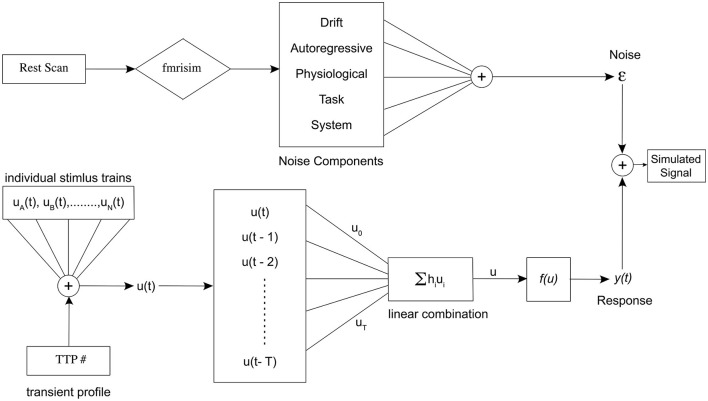
Schematic representation of the entire simulation framework. Divided into two distinct pipelines, the top portion of the figure represents the noise pipeline, and the bottom shows the response signal pipeline. Finally, they are added together to obtain the final simulated signal for a specific set of parameters.

In this section, we describe the methods and theory behind our simulation model. Figure 2 shows the entire simulation framework. Primarily, there are two distinct pipelines in our model, the realistic fMRI noise and the signal consisting of alternating event sequences, that are combined to generate the realistic brain signal.

#### 2.1.1. Nonlinear response

In order to enable the model to describe the neuronal and neurophysiological nonlinear dynamics of the human brain, we use Volterra series (Wray and Green, 1994), as initially described in Friston et al. (1998). This approach has the ability to capture ‘memory' effects; that is, it can be used for system identification where the output of a nonlinear system depends on the input to the system at all other times. It can be represented as a natural extension of the classical linear system representation and has the general form:


(1) $$\begin{array}{l} y(t)=h_{0}+\int_{R}h_{1}(\tau_{1})\cdot u(t-\tau_{1})d\tau_{1}n\\ +\iint_{R}^{}h_{2}(\tau_{1},\tau_{2})\cdot u(t-\tau_{1})\cdot u(t-\tau_{2})d\tau_{1}d\tau_{2}n\\ +\iint_{R}^{}h_{3}(\tau_{1},\tau_{2},\tau_{3})\cdot u(t-\tau_{1})\cdot u(t-\tau_{2})\cdot u(t-\tau_{3})d\tau_{1}d\tau_{2}d\tau_{3}\\ +... \end{array}$$

Mathematically, *y*(*t*) is an output signal, in our case the hemodynamic signal or the fMRI response, u(t) is the stimulus or event sequence, *h*_*n*_(τ_1_, τ_2_….τ_*n*_) is the *n*^*th*^ order Volterra kernel. For simplicity, we reduce the series to its 2^*nd*^ order, and use a causal form as derived in Friston et al. (1998),


(2) $$\begin{array}{l} y\left(t\right)=f\left(\overset{T}{\underset{0}{\int }}h_{1}\left(\tau_{1}\right)\cdot u\left(t-\tau_{1}\right)d\tau_{1}\right) \end{array}$$

where *f*(.) is a nonlinear scalar function. Expansion of *f*(.) as a McLaurin series gives us the 2^*nd*^ order Volterra series,


(3) $$\begin{array}{l} y(t)=f(0)+f(0)\\ +f^{\prime }(0)\int_{0}^{T}h_{1}(\tau_{1})\cdot u(t-\tau_{1})d\tau_{1}n\\ +f^{\prime \prime }(0)\int_{0}^{T}\int_{0}^{T}h_{1}(\tau_{1})\cdot h_{1}(\tau_{2})\cdot u(t-\tau_{1})\cdot u(t-\tau_{2})d\tau_{1}d\tau_{2} \end{array}$$

**Figure 3:**
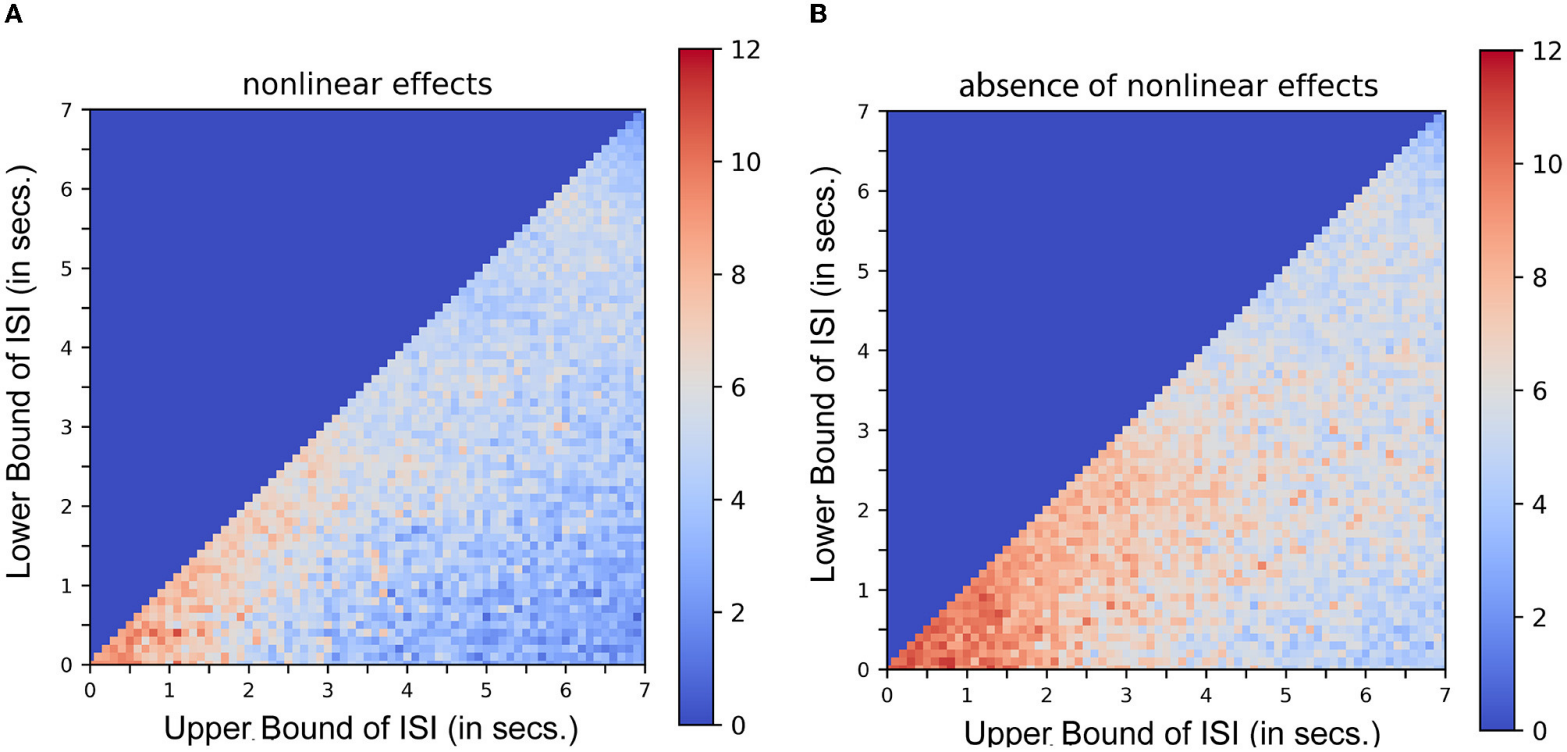
Nonlinear interactions in stimulus sequences. Integrated response over a simulated 441s single event stimulus sequence as a function of lower and upper bounds of ISI. Each dot pixel represents the magnitude of integrated response in units. **(A)** Response in presence of nonlinear effects. **(B)** Response in absence of nonlinear effects.

Equation 3 demonstrates the mathematical similarity to a 2^*nd*^ order Volterra series, with its first order kernel *h*_1_(τ_1_) as the canonical double gamma hemodynamic response function identified in a least-squares approximation or a linear regression analysis (Friston et al., 1995, 1998). The second-order kernel is a product of the first-order kernel with itself. Or in other words, *h*_2_(τ_1_, τ_2_) is replaced by *h*_1_(τ_1_).*h*_1_(τ_2_). This model can be expressed as a convolution of the stimulus function with the first-order kernel (the latent hemodynamic response function) and then expressing it as a higher-order (e.g., second-order) polynomial of itself. Figure 3 shows the results of a typical simulation. This represents the average response, integrated over a 441s stimulus train. The data were simulated based on a single event presented at different rates, the duration of the interval (or ISI) being jittered uniformly and modulated by the bounds of ISI. Each point or pixel in the space represents the corresponding average response integrated over the entire sequence. Figure 3A represents the estimated response when nonlinear effects are taken into consideration (using both the first and second-order kernels). Figure 3B represents the same in the absence of nonlinear effects (i.e., by setting the second-order kernel to zero). Contrasting Figures 3A, B, the saturation of response at or below 2s ISI in the latter case shows that nonlinearities become important at short ISIs at ~2*s* and less. These results are in agreement with what was described in Figure 3 of Friston et al. (1998). It can be noted that the average response increases asymptotically as the ISI becomes shorter which is not the case when the second-order kernels are taken into account (Figure 3). (To compare the optimality measures in absence of Volterra Kernels, refer to Supplementary Figure 1).

#### 2.1.2. Event sequence and transient temporal profile

Another issue that is important to consider for our simulation is whether the hemodynamic response itself varies with varying parameters, such as the time between events. In our modeling, we are focusing on a particular type of experimental design that has a systematically alternating stimulus sequence, such as a cue-target design, typical of studies of attention (e.g., Posner and Raichle, 1994; Corbetta et al., 2000; Hopfinger et al., 2000a). A prime question in this design context is how the stimulus-onset asynchrony (SOA) between cue and target affects the ability to deconvolve the overlapping hemodynamic responses. On the one hand, as a general principle, as the SOA becomes longer, the overlap between the cue-related and target-related hemodynamic responses is reduced, and so on the face of it, one should simply use longer SOAs (e.g., Hopfinger et al., 2000a). However, on the other hand, there are psychological, experimental, and practical reasons not to simply use very long SOAs (e.g., 10 s and over). Since these types of cognitive neuroscience studies manipulate human behavior as the goal, longer cue-target SOAs undoubtedly also affect how the subject performs (explicitly or implicitly) the task, and likely related to this, the shape of the hemodynamic response. For example, studies of working memory have shown delay period activity in the hemodynamic response that appears as an increased duration of the response (e.g., Jha and McCarthy, 2000); put another way, the hemodynamic response does not return to baseline as rapidly as it would in response to a simple sensory or motor event requiring no subsequent cognitive processing. This delay period activity can be attributed in different paradigms to cognitive functions such as attention shifting, attention engagement, memory encoding, retention, or retrieval, motor preparation, cognitive task set, and other cognitive processes (Jha and McCarthy, 2000; Hopfinger et al., 2001; Ranganath et al., 2003; Slagter et al., 2007; Noah et al., 2020). We modeled this non-linearity in our study, referring to it as transient temporal profiling (TTP), using sub-impulse functions (amplitude < 1).

The event sequence, u(t) from Equation (3) in Section 2.1.1. is modeled as follows,


(4) $$\begin{array}{l} u\left(t\right)=u_{1}\left(t\right)+u_{2}+...+u_{n}\left(t\right) \end{array}$$

where,


(5) $$u_{i}(t)=\{\begin{array}{ll} 1 & event\\ 0 & noevent \end{array},i=1,2,3...n$$

*u*_*i*_(*t*) is the event sequence for an individual event type.

In the event sequence, the occurrence of a brief event or task is represented with a 1 lasting for a duration of 1 TR step, where 1 TR has been set to 1.5s. The value of 0 is set to indicate either no event (stimulus) or the baseline activity. Throughout the study, the spacing (SOA) between two events is jittered uniformly as a function of the lower (*L*_*ISI*_) and upper (*U*_*ISI*_) bound of the time interval (for example, 2–8 s). In order to incorporate a TTP in our model related to the stimulus sequence, we modified the method proposed by Ruge et al. (2009) and implemented it to create the neural input functions for different paradigms and interval parameters. In addition to modeling the neural input function for an event as 1.0, Ruge et al. (2009) created graded amplitude profiles to mimic the preparatory processes as observed in selective attention experiments. For short delays between events, they used graded amplitudes of 1.0 and 0.66, and for longer delays, they used 1.0, 0.66, 0.66, 0.66 and 0.66. We modified their approach and further broadened it to comprehensively model more TTPs related to selective attention and working memory across different ranges of lower and upper bounds of ISI. Specifically, we adapted the amplitudes of their graded profiles (1.0 for event-related activity and 0.66 for preparatory activity) but placed them in a different sequence and order of delays. Thus, TTPs 1 and 2 were created following their method, and TTPs 3–6 were created by modifying them as described below. This modification was important to accurately emulate the different cases of preparatory activity during selective attention or working memory experiments that use non-randomized alternating sequences of stimuli (e.g., cue-target designs).

**Figure 4:**
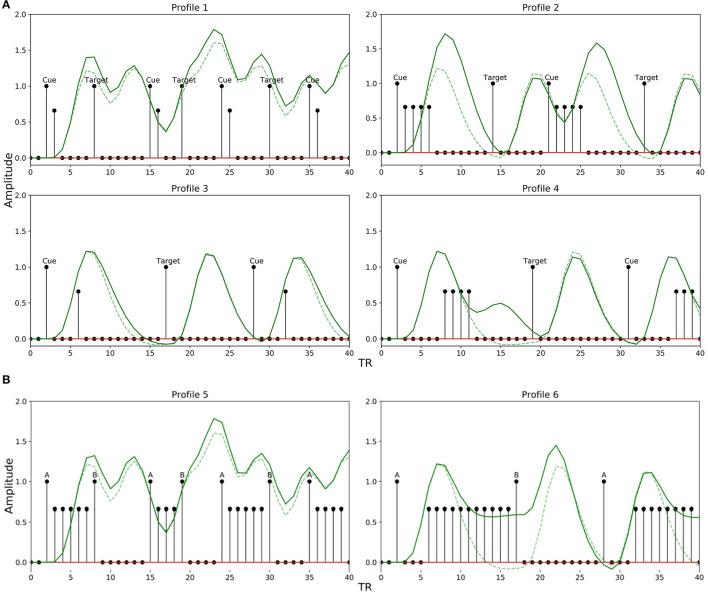
Transient temporal profiles (TTPs) used in the simulation. (Solid Green Line: BOLD response in the presence of sub-impulse functions; Dotted Green Line: BOLD response in the absence of sub-impulse functions). **(A)** Profiles 1–4 was designed for an attentional control paradigm. **(B)** Profile 5 and 6 was designed for a working memory paradigm.

We explored a limited range of patterns theoretically possible for experimental designs common in attention and working memory-related studies, modeling the TTPs accordingly. The different profiles are shown in Figure 4. Profiles 1–4 are implemented to emulate paradigms related to attentional control, while 5 and 6 are for working memory. Each profile is used to mimic neural activity throughout the entire interval period. The sub-impulse functions (amplitude < 1) are placed in between the events to represent delay period activity. Note that the BOLD response in the presence of TTPs (Figure 4, solid green lines) is different from when modeled just using the stimulus functions (Figure 4, broken green lines). This maintenance of BOLD activity raised above baseline during the delay period not only plays a significant role in non-linear interactions as discussed in Friston et al. (1998) but is also crucial in accurately modeling the activity during the interval period- as reported in Medendorp et al. (2006), Sylvester et al. (2008), and Liu et al. (2016).

**Figure 5:**
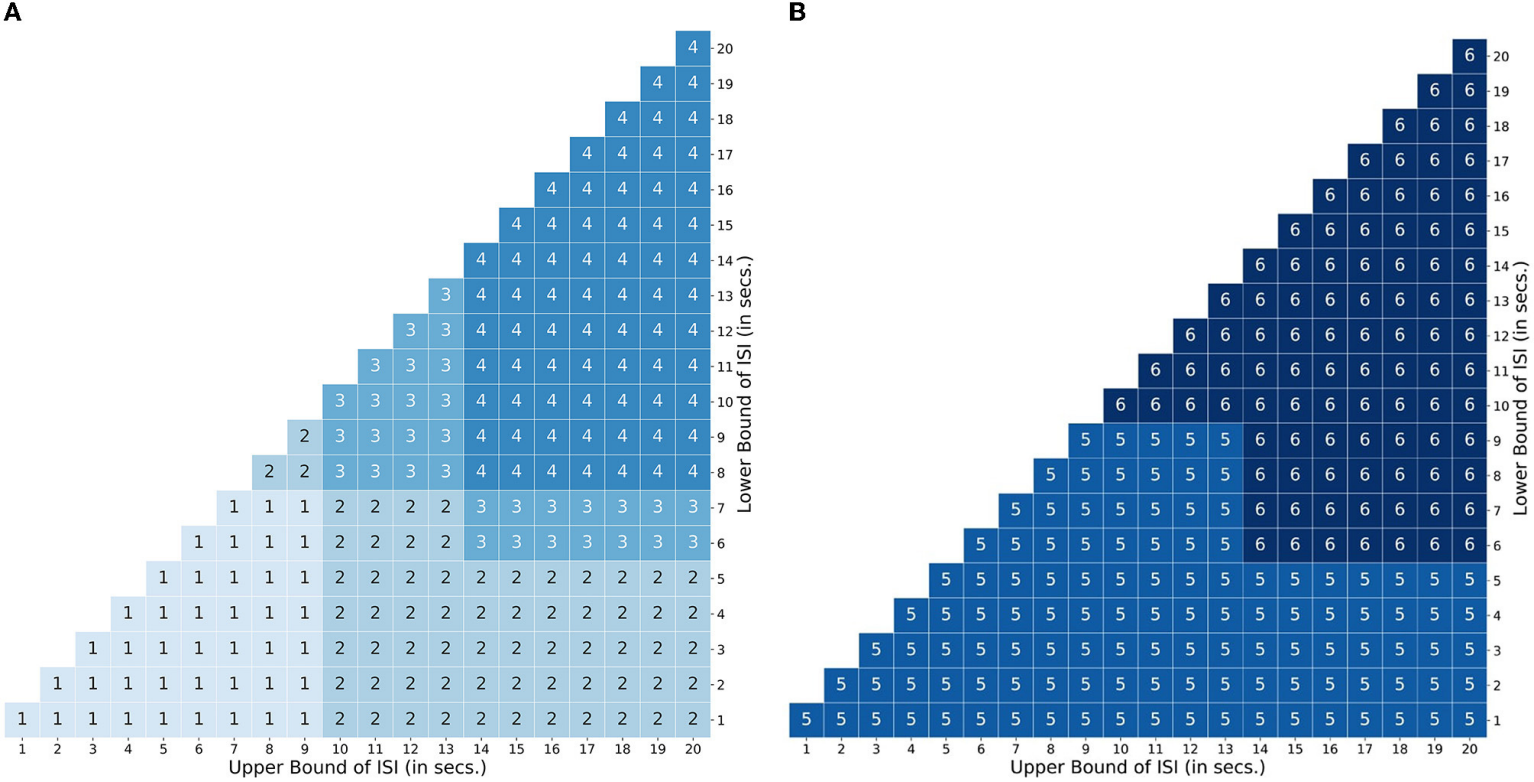
Transient Temporal Maps (TTPs). TTP map encompassing the space defined by the bounds of the inter stimulus interval. **(A)** Usage map of profiles 1 - 4 designed for an attentional control paradigm. **(B)** Usage map of profiles 5 and 6 for a working memory paradigm.

For attentional control experiments, as discussed earlier, the event sequence is designed as cues followed by targets. In order to account for the possible variations in the BOLD signal during the interval between a cue and target related to attentional control, Profile 1 was designed with two amplitudes [1, 0.66] lasting 2 TR steps for short delays between a cue and target (short *L*_*ISI*_ and *U*_*ISI*_). Similarly, Profile 2 has amplitudes [1, 0.66, 0.66, 0.66, 0.66] lasting 5 TR steps, and is used for intervals having short *L*_*ISI*_ but long *U*_*ISI*_. Profiles 3 and 4 are analogous to 1 and 2, respectively, with the only exception being in the case of longer *L*_*ISI*_ designs. The brief delay period in Profiles 3 and 4 between stimulus onset and maintenance-like activity lasting for *L*_*ISI*_ / 2 TR steps, is intended to mimic a subject's relaxation period prior to preparatory activity when the subject estimates a somewhat definitive long interval (longer *L*_*ISI*_) before the upcoming target. Figure 5A shows how different TTPs for attentional control are implemented across the map of *L*_*ISI*_ and *U*_*ISI*_.

For experiments related to working memory, Profiles 5 and 6 were constructed to account for ongoing maintenance-like activity. Profile 5 was designed with varying amplitudes of [1,0.66.0.66…...until next event], to model an extremum of the constant maintenance of BOLD signal between two events when they are spaced close to each other (short *L*_*ISI*_ and *U*_*ISI*_). Profile 6 designed for intervals having longer *L*_*ISI*_, is similar to 5, with an exception of the inclusion of a brief delay in the onset of maintenance-like activity. Figure 5B describes the usage of TTPs 5–7 for the case of working memory across the map of *L*_*ISI*_ and *U*_*ISI*_.

Most importantly, for all the TTPs, the exact values were chosen following the approach of Ruge et al. (2009). Also, the duration of the brief delay period in TTP Profiles 3, 4, and 6 was deliberately chosen as *L*_*ISI*_/2, to model the situations that may arise in cue-target designs with longer delays such as subject delaying the engagement of the cognitive operations of interest until they decide to, or the time it takes for the brain to ramp up to reach maintained levels of activity. We retained the primary profiles because other similar scenarios produced highly similar parameter estimates and optimality results (see Supplementary Figures 2–4).

#### 2.1.3. Noise source

**Figure 6:**
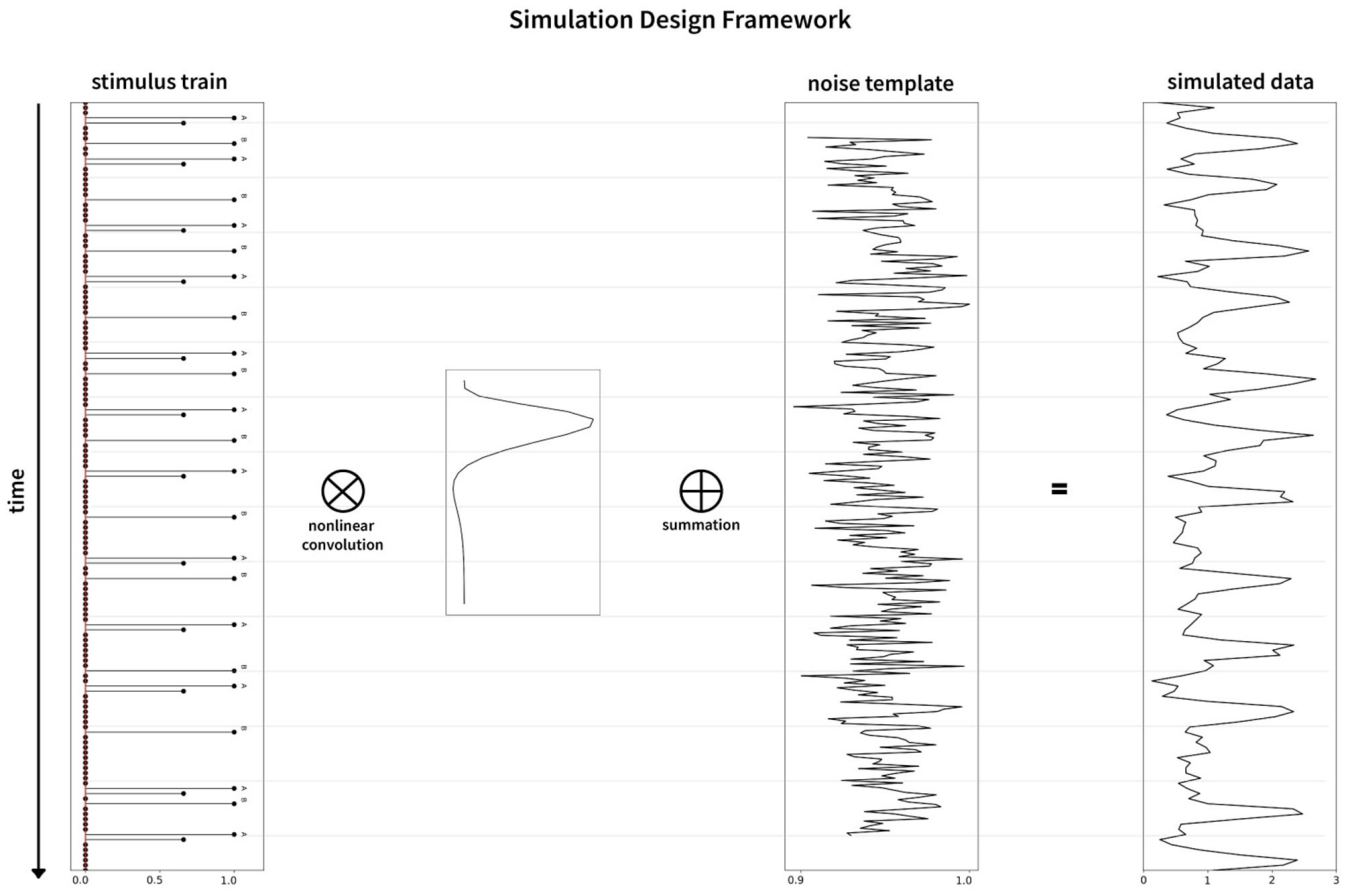
Graphical representation of the simulation framework. The stimulus train **(left)** is convolved nonlinearly with the hemodynamic response function (inset between stimulus train and noise template), followed by addition of the noise template **(middle)** to generate the simulated data **(right)**.

Noise in the fMRI environment consists of physiological noise related to cardiac and respiratory activity, head/body movements, system and task-related noise, drift, and autoregressive/ moving average (AMRA) noise related to the machine. We used fmrisim (Ellis et al., 2020) to extract the different noise parameters and to generate a similar noise template from a real fMRI dataset. For our analysis, we used the publicly available dataset (Bejjanki et al., 2017), (http://arks.princeton.edu/ark:/88435/dsp01dn39x4181). We used a rest run data from the dataset in order to estimate and generate the noise to be included in the model. Finally, the response generated as described in Section 2.1.1 was combined with the noise template to obtain a realistic brain response for a particular experiment. The noise pipeline in the simulation is presented in the upper half of Figure 2. The graphical representation of the simulation framework including the generation of the BOLD sequence is presented in Figure 6. In addition, we found a significant effect of the noise source in modeling the responses as well as the optimality measures (see Supplementary Figure 5). Our statistical models fit significantly better when using realistic fMRI noise as compared to other types of noise.

### 2.2. Parameterizing design optimality

To parameterize the optimality of our simulated designs and estimate the underlying signal, we implemented the statistical methods used in previous works (Dale, 1999; Josephs and Henson, 1999; Wager and Nichols, 2003; Henson, 2007; Kao et al., 2009). It involves generating a predicted response by convolving the hemodynamic response function with the stimulus sequence (Figure 6), which is then compared to the actual simulated response using General Linear Models (GLM). To deal with the autocorrelational nature of the fMRI signal, we used a prewhitening approach to generate the covariance matrix of the parameter estimates for GLM. This is discussed in detail in the Supplementary material.

### 2.3. Simulations

In this section, we consider alternating event-related fMRI sequence simulations with two event types namely a cue and a target in a sequence, where a target is always preceded by a cue. The magnitude of TR was set to 1.5s and the generation length was set to 294 TRs (equal to the duration of the noise template from fmrisim). The contrast matrix C was chosen to be [1 0], due to our primary interest in the cue-induced activity (in attention-related experiments), its convolution pattern with other signals, and the interval between a cue and its successive target. In addition, we used the canonical HRF, a mixture of two Gamma functions that elicit a peak at around 5s followed by a subsequent undershoot (SPM12, http://www.fil.ion.ucl.ac.uk/spm). The duration of the HRF was 30s where the response returned to baseline after 16 s. All the reported simulations included the second-order nonlinear model as derived from Equation 3.

#### 2.3.1. Simulation 1

We first investigated how ISI influenced design optimality. We generated a fitness landscape of detection power and estimation efficiency by exploring every combination of *L*_*ISI*_ and *U*_*ISI*_ from 1s to 20s with increments of 1s. The efficiency measure for each point in the fitness landscape was derived by taking the mean optimality as the population reference of 100 random event sequences. The ISI was uniformly jittered between the bounds of *L*_*ISI*_ and *U*_*ISI*_. The magnitude of activation of cues and targets (or events A and B - whose amplitude were set to 1 in the stimulus sequence) were set to be equal to each other i.e., equal to 1. Simulations were separately carried out for attentional control TTPs and working memory TTPs.

#### 2.3.2. Simulation 2

Past work has suggested that in any event-related fMRI experimental design with a rapid presentation of events, the introduction of null—events at random positions can ameliorate the efficiency of a design (Friston et al., 1999; Josephs and Henson, 1999). Null events can be considered as non-occurrence of an event that should have occurred in a given generated sequence. In this simulation, we set a certain proportion of targets to be null events, and we call them “null-targets,” and thus the null targets are the non-occurrence of a target when the target would normally occur in the design. Since one cue followed by one target constitutes a “trial,” a cue followed by a null target is referred to here as a “partial trial.” We varied the proportion of partial trials in a sequence from 0% to 50%. For instance, if a sequence is made up of 100 trials, a proportion of 10% partial trials would mean 90 complete trials and 10 partial trials, or in other words, any 10 random cues will not be followed by their respective targets. Along with that, we further varied the *U*_*ISI*_ from 2 to 20 with an increment of 2, with *L*_*ISI*_ held constant, to explore how these parameters affect the optimality of a design. In a similar fashion to Simulation 1, the magnitude of activation of all the events was set as equal to each other. This simulation was carried out in the paradigm of attentional control only (TTP 1–4).

### 2.4. Computation time

All simulations were coded in Python 3.7.1 and run on an Apple MacBook Pro 2017 with a 2.3 GHz Intel Core i5 and 8GB 2133 MHz DDR3 RAM. For a generation size of 294 TRs, Simulation 1 took 2 min and 27 s to complete. Simulation 2, having limited iterations, took less than a minute. On another note, when simulation 1 was carried out separately with a longer generation size of 660 TRs, required ~18 min to conclude.

## 3. Results

### 3.1. Simulation 1

**Figure 7:**
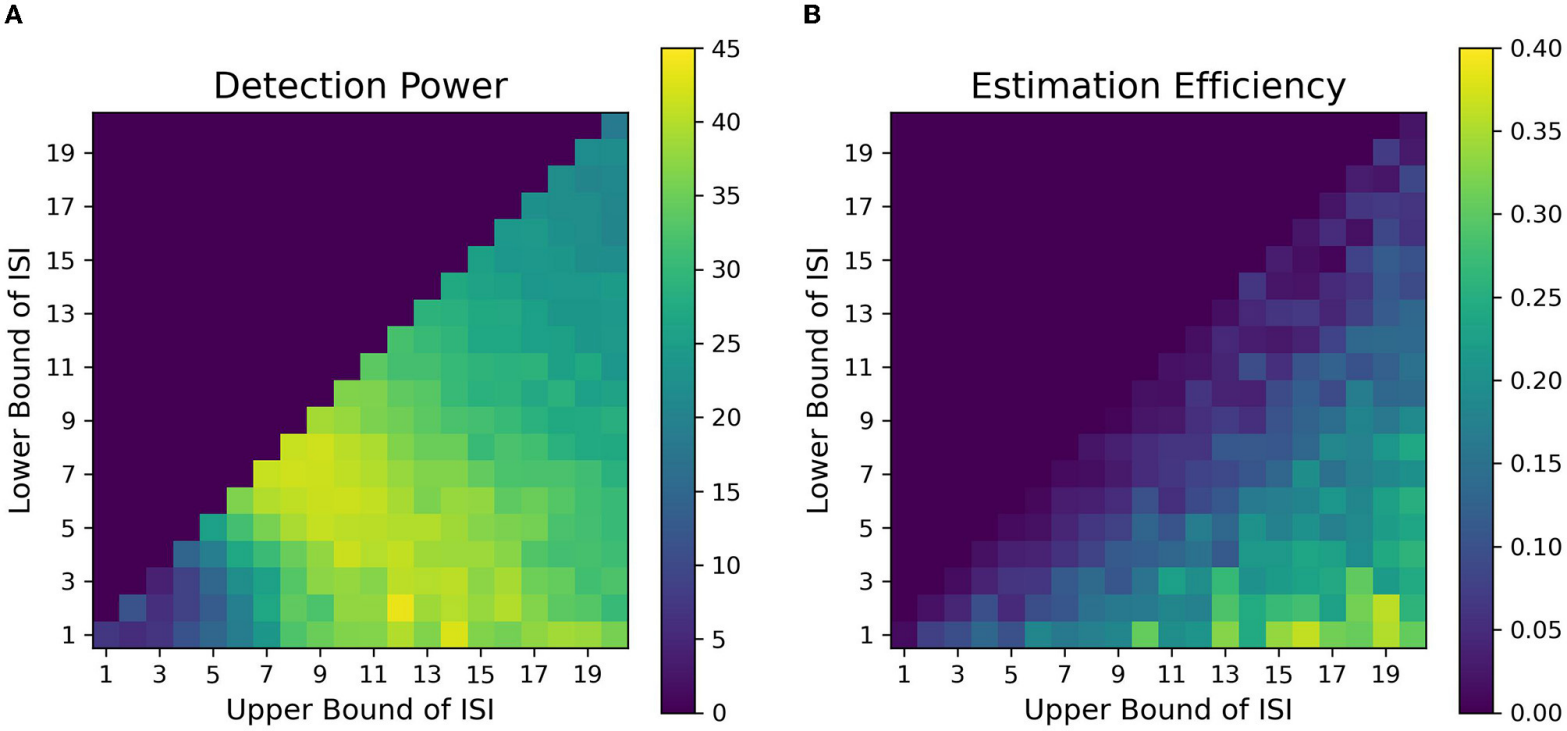
Fitness landscapes for Simulation 1–*Attention*. Each pixel in the landscape represents the corresponding measure of optimality as a function of lower and upper bounds of ISI for attentional control transient profiles (TTP 1-4). **(A)** Detection power. **(B)** Estimation Efficiency.

The fitness landscape for the attentional cueing paradigm in Figure 7A shows the detection power as a function of *L*_*ISI*_ and *U*_*ISI*_. Each pixel in the landscape represents the corresponding detection power as a function of a given *L*_*ISI*_ and *U*_*ISI*_. For shorter ISIs ~1 − 2*s*, the results depict a sharp drop in detection power. Further, we find that the maximum power to detect a signal is when both the bounds of ISI are around 5-15s (*L*_*ISI*_ and *U*_*ISI*_ respectively), as observed in Figure 7A. These results replicate the findings of Wager and Nichols (2003) (their Figure 6, simple nonlinear assumption) and Josephs and Henson (1999) (their Figure 2A, alternating DE). We have also observed the effects of TTPs separately on the optimality of design sequences. In general, TTPs of various forms contribute toward increasing the entropy of the signal thus aiding the detection of peaks in modest ISI ranges (LISI 2-3s, UISI 3-6s, Supplementary Figure 6).

Figure 7B provides the estimation efficiency as a function of ISI bounds for the attentional cueing simulations. Results as expected, tend to peak at low *L*_*ISI*_ and high *U*_*ISI*_. This suggests that the higher the bounds of jitter of interval is, the better the shape of the HRF is retained in the response signal; that is, there is less overlap in the HRFs, leading to less distortion. In order to visualize the effects of the optimality measures across parameters, we investigated the individual BOLD time courses. Figure 9 compares the different simulated responses. Note that region II (*L*_*ISI*_~5, *U*_*ISI*_~9) has the highest detection power. This can also be corroborated by its corresponding time course, which has clear variations in the signal and the individual hemodynamic response peaks are resolvable thus optimizing the detection power. On the contrary, the poor shape of individual responses reduces estimation efficiency to a large extent.

On another note, region III ((*L*_*ISI*_~3, *U*_*ISI*_~19) has the most optimal HRF estimation efficiency. In Figure 9, at the top left is the underlying hemodynamic response function (canonical double gamma HRF) of all simulations. Precise similarities can be observed between the shape of the underlying response function and that of individual responses in the time course of region II, thus maximizing estimation efficiency. It can be argued that having such distinctive and resolvable peaks of the individual responses for regions III and IV, the detection power should also be high. But it is also the case that having extremely long intervals between stimuli reduces the total number of trials in a particular sequence (since the total time is limited), thus indirectly affecting statistical power and detection of the signal. It can also be noted that as events are spaced close together in time as defined by parameters of region I, the time course shows minimal variation in the signal, thus containing no meaningful information to either detect or estimate the response. On a side note, if the events were randomized and rapidly presented as per the parameters at region I, as performed in Burock et al. (1998), the time course would have had much better estimates about detection and estimation. Since the alternating designs cannot exploit the power of randomization, rapid presentation of stimuli being challenging is a less sought-after choice.

### 3.2. Simulation 2

In Simulation 1, we showed the influence of ISI on the optimality of a design sequence. In this Simulation 2, we further assess the effect of both ISI and the proportion of partial trials (null events) on design optimality. In this design, partial trials were randomly distributed, and their proportion was varied in a sequence. Figures 10A, B, respectively, show how detection power and estimation efficiency are modulated by the inclusion of partial trials and parameters of ISI. The extended delay interval in partial trials now provides an added baseline in a design that directly influences the optimality. From Figure 10A, we see that inclusion of partial trials primarily affected the detection power at shorter ISIs. As the *U*_*ISI*_ increased, with *L*_*ISI*_ held constant at 2, the detection power for all partial trial proportions converged. This pattern reflects that individual responses to events that are close together in time, overlap with each other, thus making it difficult for the predictors in the design matrix to explain the variance in the BOLD response. Thus, the inclusion of partial trials effectively spaces out events (and introduces a larger jitter in the ISI), which reduces overlap between individual responses, and as a result, increases the detection power. Additionally, this effect diminishes as the bounds of the jittered delay interval are increased, to a point (~14*s*) where the overlap is minimized due to the spacing of events. These results are in agreement with Friston et al. (1999) (their Figure 6B, dotted line).

The estimation efficiency measures, shown in Figure 10B, depict an asymptotic relationship between efficiency and ISI bounds for all proportions of included partial trials. It can also be seen that there is a benefit even with only 10% partial trials included in the design sequence, compared to having no partial trials. These findings were further extended and corroborated by running different simulations that considered greater fixed *L*_*ISI*_ (Supplementary Figures 7, 8).

## 4. Discussion

The aim of the present simulation study was to identify the optimal design parameters for event-related fMRI experiments that include alternating stimuli (e.g., cue-target designs). Our analyses included a simple but realistic model of nonlinearity and the noise profile of BOLD signals in fMRI. Our simulations considered the roles of (i) cue-to-target ISI (ii) size of the ISI jitter (iii) effects of nonlinearity that is related to experimental factors, such as whether the shape of the HRF would be expected to be different as a function of, for example, maintenance like activity during working memory or attention, and (iv) the proportion of partial-trials i.e., cues not followed by targets.

**Figure 8:**
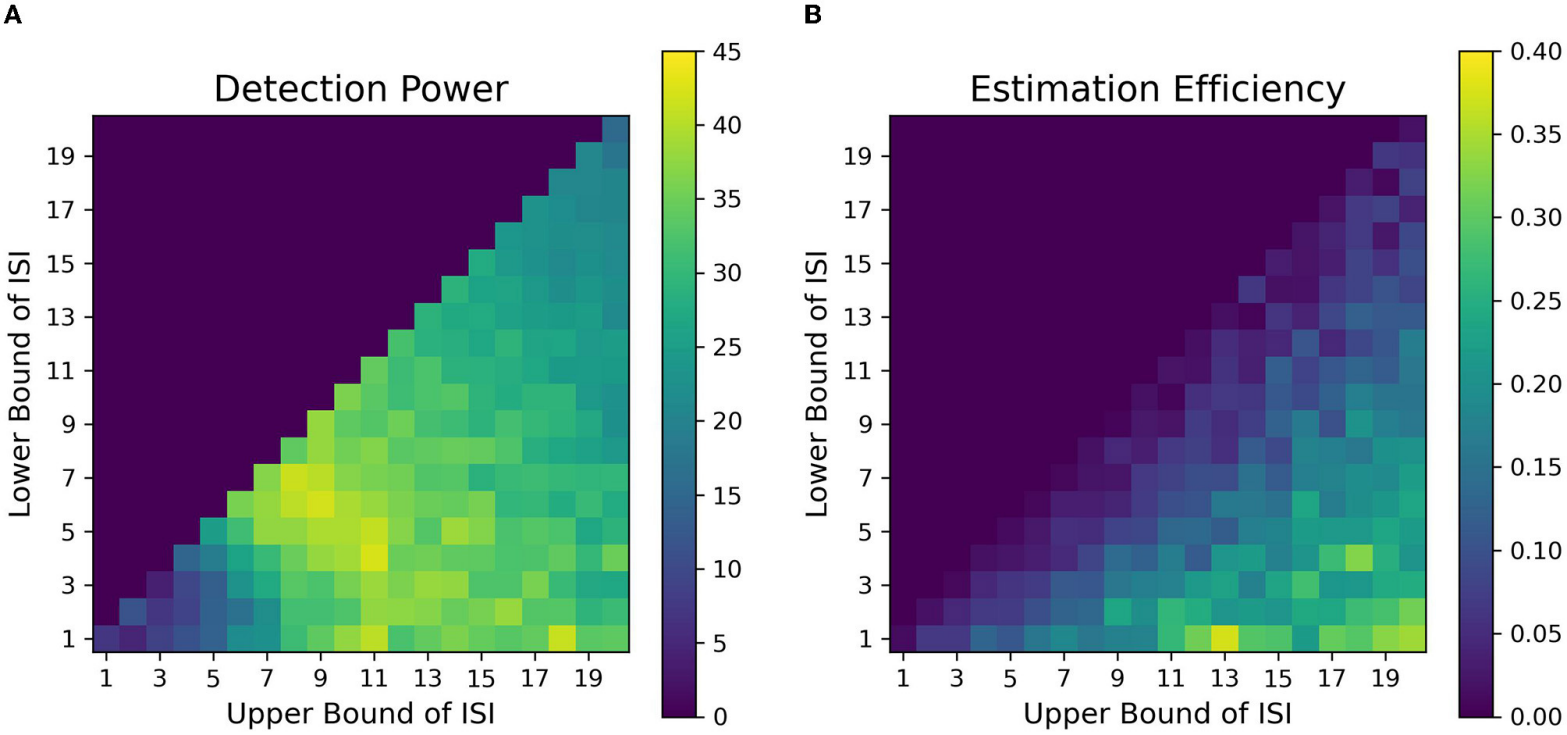
Fitness landscapes for Simulation 1–*Working Memory*. Detection power **(A)** and estimation efficiency **(B)** as a function of lower and upper bounds of ISI for working memory transient profiles (TTP 5 & 6).

We found that contrary to typical assumptions in fast, randomized event-related designs (Burock et al., 1998, at short ISIs around 2–4 s, detection power falls off dramatically in alternating designs. This is likely because of the saturation of the signal when events are placed close to each other in time in an alternating sequence. When saturation occurs, the individual signal peaks for each event overlap with each other to large extents, thus making it difficult to deconvolve one from the other. In addition, at short ISIs, the sluggish time course of the HRF is too slow to capture the high-frequency components of the underlying neural processes associated with the closely placed events. We found that the optimal detection power for alternating event-related fMRI designs was obtained at intermediate ISIs having *L*_*ISI*_ and *U*_*ISI*_ of around 5-15s. Such an ISI space, with a mean ISI of around 9 s, gives us an inter-event interval (interval between events of the same type, e.g., cue-to-cue) of around 18 s, which closely resonates with the dominant frequencies of the assumed HRF. Finally, when the duration of ISI is increased over 16 s, we observe a sharp drop in detection power. This can be attributed to weighing against the number of allowable trials with increasing ISI since the total duration of the experimental sequence is finite in our simulations; this was done because we want to investigate real-world parameters, which include limitations of time in the scanner. Thus, to consistently maintain high detection power for finite-length experiments, researchers should avoid the asymptotic bounds of ISI. We found similar ranges of optimal parameters of detection power for our simulations of designs in working memory tasks (Figures 8A, B).

On the contrary, we found that the highest estimation efficiency can be achieved with maximum jitter during the delay period i.e., with the lowest *L*_*ISI*_ and highest *U*_*ISI*_. Our results indicate that estimation efficiency starts to peak at ~16*s*. Since our assumed HRF was modeled to be 16s long before returning to baseline, a jittered ISI having low *L*_*ISI*_, and *U*_*ISI*_ greater than 16s was better able to estimate the variance in the response signal at each corresponding time point of the HRF as dictated by our assumed basis functions.

**Figure 9:**
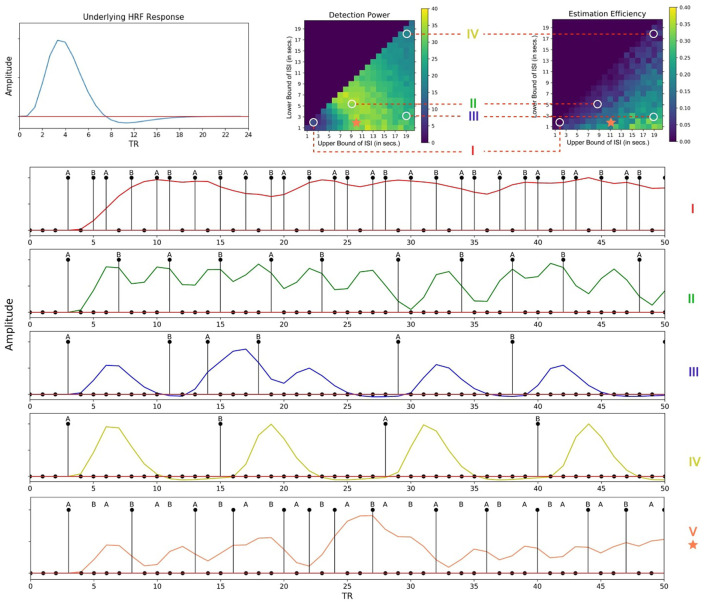
Individual time courses of hemodynamic responses corresponding to different regions of the fitness landscapes. **(Top Left)** Underlying hemodynamic response function of the time courses. **(Top Right)** Different regions of the fitness landscapes. **(Bottom)** Corresponding time courses; (I)- [*L*_*ISI*_~2, *U*_*ISI*_~3], (II)- [*L*_*ISI*_~5, *U*_*ISI*_~9], (III)- [*L*_*ISI*_~2, *U*_*ISI*_~19], (IV)- [*L*_*ISI*_~18, *U*_*ISI*_~19]; Region V [*L*_*ISI*_~2, *U*_*ISI*_~10], denoted by an asterisk would be ideal for experiments optimized for both detection power and estimation efficiency. (Note: These time courses have been simulated without any noise to better display the details of the individual responses). This figure has been updated for better readability and to fix annotation errors.

However, the key question still remains, as, what are the best parameters for optimal detection and estimation of a brain signal? Past studies (Liu et al., 2001; Wager and Nichols, 2003), have demonstrated that there is an orthogonal trade-off between detection power and estimation efficiency, i.e., one cannot optimize both at the same time. As discussed in detail in Liu et al. (2001), optimal detection power is achieved when the predictors of the design matrix are orthogonal, or in other words, in a block design. On the contrary, optimal estimation efficiency is achieved with maximum variance in the stimulus sequence (Equation 5). Therefore, maximum detection power comes at the cost of minimum estimation efficiency and vice versa. In agreement with previous studies, we found completely opposite regions in the space of parameters where each optimality criterion is optimized for a certain event sequence (Figures 7, 8). Through our design framework, it is evident that when events are placed close to each other, signals overlap and add up thus making it easier to detect a change in activity and achieve maximum detection power (Region I, Figure 9). However, the problem with such a design is the lack of information about the shape of an individual response thus resulting in the lowest estimation efficiency. On the flip side, maximum estimation efficiency can be achieved by inducing longer and jittered delay intervals between stimuli (Region III, Figure 9) so that the individual responses do not overlap. Despite the obvious shortcoming of this inverse relation, it should be noted that the apportionment of detection power and estimation efficiency is completely dependent on the specific aims of the experiment. Designs that maximize detection power are ideal for experiments that intend to establish which region(s) in the brain is active in response to a specific event. On the other hand, designs that boost estimation efficiency is ideal for experiments that aim to characterize the shape of the hemodynamic response in a prespecified region of interest in the brain. Nevertheless, we propose, at a cost to both, an ISI of around *L*_*ISI*_ = 2*s* and *U*_*ISI*_ = 10*s* would be ideal to optimize both criteria as demonstrated in Figure 9 with a star marker.

**Figure 10:**
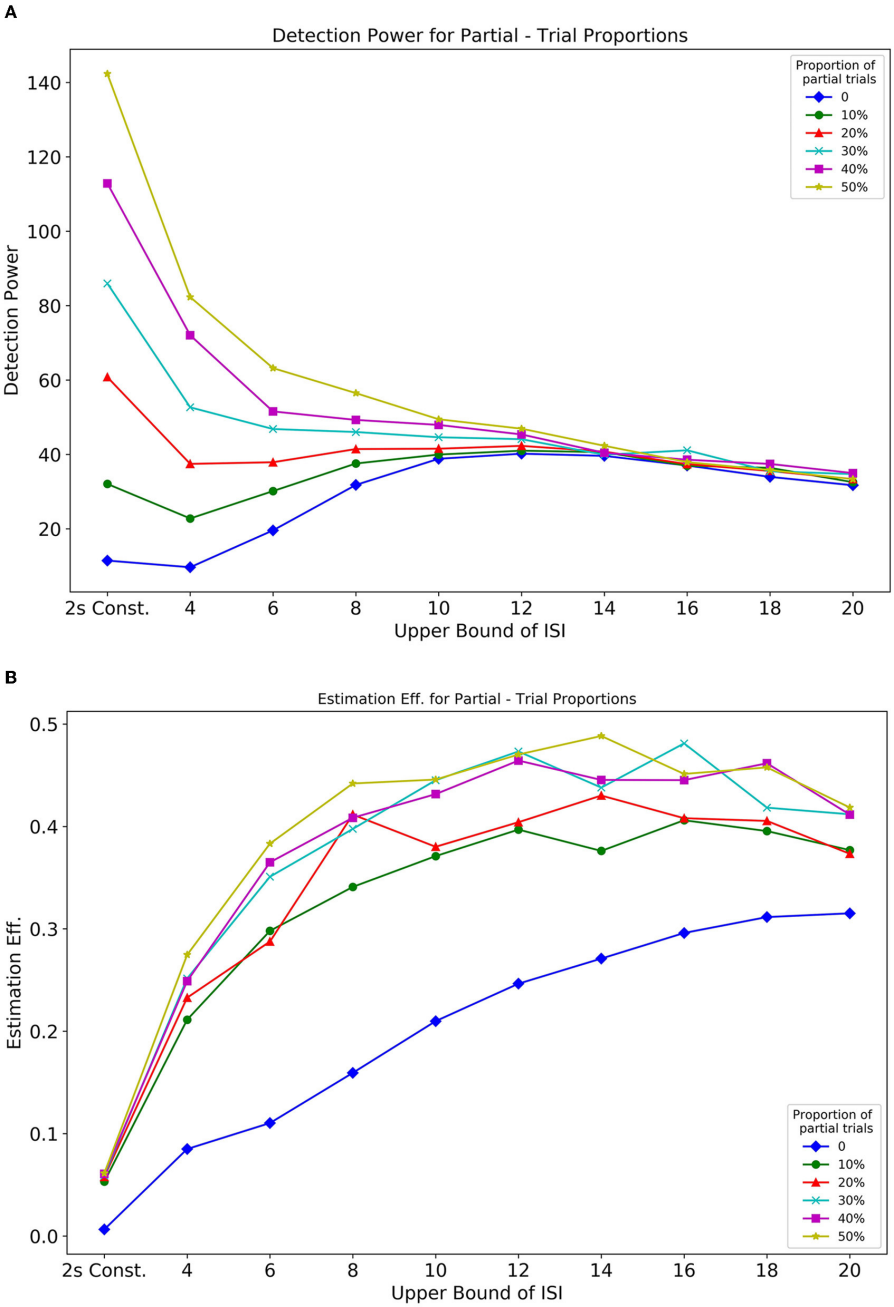
Optimality measures from Simulation 2 Optimalities are as a function of the proportion of partial trials and ISI. **(A)** Detection power. **(B)** Estimation efficiency.

In addition to how the cue-to-target ISI and its jitter influence design optimality, we also demonstrate the relation between the proportion of null events and the optimality of a design. Our results indicate how null trials can benefit a design in terms of detection power only at shorter ISIs whereas its effect on estimation efficiency is significant throughout the entire parameter space even when a mere 10% of all trials are set as null (Figure 10B). By varying the upper bound of ISI with the lower bound kept fixed at 2, we find the increase in detection power to be directly proportional to the ratio of null trials and this effect diminishes beyond 10s. Conversely, it is effective in estimation efficiency throughout the variation of the upper bound of ISI. This relation is directly proportional and somewhat monotonic up to 8s followed by an asymptotic course. Often, it has been debated in the past that null events or no-go events, can introduce bewilderment, failure of anticipation of the next event in the subject, and oddball effects. From the design perspective, this would mean a sudden truncation of the current trial. This would prompt several different brain networks to activate, which might lead to misleading data. As a cautionary note, partial trials should be followed by events not related to the experiment describing the end of a certain trial. For instance, a note on the screen saying, “END OF TRIAL,” or a certain sound pre-instructed to the subject that would mean the end of that current trial.

Apart from the design parameters, it is also important to discuss the novel factors of our simulation study that facilitate the modeling of BOLD signals. It can be noted that when events occur closely in time, there is strong evidence that nonlinear effects predominate (Friston et al., 1999; Wager et al., 2005). The optimization strategies developed in the past to evaluate and select optimal parameters for fMRI designs, though efficient, were based on the limited assumption that the convolutional nature of BOLD signals is linear. Therefore, by assuming a linear response (Dale, 1999; Liu and Frank, 2004) or an oversimplified nonlinear model (Wager and Nichols, 2003; Kao et al., 2009), past studies do not predict an accurate and reliable model of the saturation of the signal. In addition, their assumed noise models—such as temporally uncorrelated white noise, the addition of a constant nuisance parameter, or a stationary noise model—were oversimplified representations of the noise in real data. Contrary to previous studies, we demonstrate the functional and statistical significance of utilizing a more realistic fMRI noise source as opposed to random noise (Supplementary Figure 5, improved GLM fit for fMRI noise models). Similarly, we demonstrate the contribution of nonlinear interactions (Volterra kernels) and transient states between events (Supplementary Figure 6). These minute interactions must be taken into account in order to precisely estimate the optimal parameters of any event sequence. The integration of practical BOLD signal characteristics and psychological task parameters in our work yields highly accurate results and in turn enhances the existing modeling procedures.

Note that most of the assumptions and models that have been discussed in this work are based on the investigation of the same voxel. That is, the effects of different types/classes of stimuli were assumed to affect all voxels in the same fashion. In reality, say for an attentional cueing experiment, it is not the case that a cue and target will elicit the same degree of response in any given voxel, because the physical stimuli themselves may be very different (e.g., a central arrow or even auditory cue vs. peripheral visual field target stimulus), and the perceptual and cognitive activity evoked may be in quite different brain networks (e.g., attention control networks vs. sensory networks). Thus, the work we present represents the extreme case when both a cue and target activate the same voxel with equal intensities. It models the most extreme scenarios for the convolution of signals, that is when the consecutive conditions evoke identical responses, which if close together, can lead to significant overlap. We present our results from the perspective of the cue-evoked response, that is, what is the detection power or estimation efficiency of the cue-evoked response? The results will be different if the target and cue were modeled as dissimilar responses. A stronger target response would be unfavorable to cue optimality and vice-versa. These conditions have been simulated, and the results can be found in the Supplementary Figures 6, 7.

Our work helps mitigate but does not eliminate, the challenges of deconvolution of overlapping hemodynamic responses in many common experimental situations using non-randomized sequences. The field of cognitive neuroscience has developed efficient routines to achieve high statistical efficiency in fMRI designs. However, in many cases, studies that use non-randomized alternating event sequences have not considered the problems discussed here (see however, Woldorff et al., 2004). This leads to severe overlapping of adjacent brain responses which can potentially lead to poor efficiency. Our work discusses different parameters and their limits that are considered for designing such alternating event sequences. By carefully choosing the design parameters for an alternating sequence, such overlapping brain responses can be deconvolved to a great extent. Our results serve as a reference to cognitive neuroscientists who intend to develop optimal designs for alternating sequences in their research.

## 5. *Deconvolve*: an fMRI deconvolution toolbox as python module

The methods presented in this work have been compiled into an open-source Python toolbox called *deconvolve*, which can be found at https://github.com/soukhind2/deconv. This toolbox supports a wide range of design parameters, including event durations, ISI bounds, jitters in the interval between events, and nonlinear interactions between events (Friston et al., 1999). Additionally, it includes tools for modeling various HRF functions to construct time courses. It can generate diverse stimuli (multiple cues and targets as well as null events), ISI jitters (uniform, exponential, or stochastic), and response amplitudes for each event. In a future release, the toolbox will support versatile functions to manipulate trial-to-trial variability (Abdulrahman and Henson, 2016) including specifiable spatial covariance in neighboring voxels (Mumford and Nichols, 2006; Wu et al., 2021) and scan variability (McGonigle et al., 2000).

This toolbox extends the limitations of existing Python and MATLAB modules/toolboxes. Contrary to them, *deconvolve* uses nonlinear interactions, an fMRI noise source that is more realistic, inter-interval transient states (TTPs), unequal response amplitudes, and versatile timing parameters to model event sequences. Furthermore, it is specifically designed to simulate alternating event sequences in addition to random sequences. We provide a seamless framework to cognitive neuroscientists, who wish to simultaneously modulate several factors of the BOLD signal and standard timing parameters to initiate and design their alternating event-related fMRI experiments.

## 6. Conclusion

In this simulation study, we investigate the optimal design parameters for event-related fMRI studies where stimuli occurring closely in time may result in overlapping BOLD signals. We focused on a particularly challenging experimental design type, where alternating event sequences (non-randomized sequences, e.g., cues-targets) result in poor results for many common analytic approaches. We present a model structure that provides insight into how the performance of an alternating event sequence varies based on different experimental design parameters. Through our assumption of a practical nonlinear model for the refractoriness of the hemodynamic response, implementation of a realistic noise model of fMRI data–and taking into account the various transient temporal profiles–we have identified the design parameters for reliable estimates of optimality for alternating event-related fMRI designs.

We found that long ISI designs are less efficient in terms of detection power, although they work well, as expected, for characterizing the hemodynamic waveform (i.e., efficiency). As expected, we found that for a particular design sequence, it is difficult to simultaneously optimize both detection power and efficiency. We included simulations showing how the inclusion of null events at different ISIs can optimize alternating designs; in alternating event-related fMRI designs with rapid presentation of stimulus, the inclusion of null events (partial trials) is critical for fast rates (shorter ISIs) as it increases the detection power of a design many folds. In addition, we found a direct relationship between the proportion of null trials and an increase in estimation efficiency. We also introduced transient temporal profiles using graded stimuli, which is a novel factor to characterize the maintenance-like activities in an event-related fMRI experiment and should be considered to optimize the design of an alternating event-related fMRI experiment. Their use made our framework more practical by factoring in the pivotal maintenance-like activity that is usually present in alternating event-related fMRI designs and influences their optimality.

Our aim here was to provide some additional information that will permit cognitive neuroscience researchers to develop optimal designs for many common experimental designs used in fMRI. Nonetheless, future developments for event-related fMRI studies using alternative event designs will need to focus on (i) multiple trial type designs and their contrasts, where there are multiple cues and targets; (ii) assessing parametric characteristics of the design matrix, from a mathematical point of view, and (iii) the different types of jitters such as exponential, fixed, and dynamic, stochastic, and so on.

## Data availability statement

The datasets presented in this study can be found in online repositories. The names of the repository/repositories and accession number(s) can be found at: https://github.com/soukhind2/deconv.

## Author contributions

SD and WY developed the code for the simulations and toolbox. SD, MD, and GM contributed to the conception and design of the study, contributed to the writing of the manuscript, and read and approved the submitted version.
