# Supplementary material for: Optimizing cognitive neuroscience experiments for separating event- related fMRI BOLD responses in non-randomized alternating designs
Source: Front Neuroimaging. 2023 Apr 17;2:1068616. doi: 10.3389/fnimg.2023.1068616 (PMC10406298; doi:10.3389/fnimg.2023.1068616)
Supplement: Supplementary file 1 [file Data_Sheet_1.PDF]

frontiers

Supplementary Material

1 PARAMETERIZING DESIGN OPTIMALITY

We used the General Linear Model (GLM) for parameterizing the optimality of a design sequence, as shown in Equation S1,

$$Y = X\beta + \epsilon, \epsilon = N(0, \sigma) \tag{S1}$$

where Y is the $N \times 1$ voxel-wise BOLD time series obtained from simulation, X is the $N * k$ design matrix (for k events) which represents the expected response, β is a $k + 1$ vector of coefficients pertaining to the response amplitude for each condition in X and ϵ, the normally distributed error term $N(0, \sigma)$.

The efficiency of estimation is inversely related to the variance of the parameter estimates. Assuming independent errors, the unbiased estimate of the parameters is given by the least-squares estimation,

$$\hat{\beta} = (X'X)^{-1}X'Y \tag{S2}$$

However, fMRI noise shows strong evidence of significant temporal autocorrelation, so the errors are dependent and correlated. So, we used a prewhitening approach so that the parameter estimate changes to,

$$\hat{\beta} = ((KX)'(KX)^{-1})(KX)'KY, \tag{S3}$$

where K is a decorrelating matrix such that KVK' is the identity matrix and V is the correlation matrix of errors. We calculate the prewhitening matrix K by solving the initial regression equation S1 to get an estimate of the residuals. This was followed by fitting an AR model to the residuals to find the autocorrelation coefficients and use them to form the correlation matrix V. Finally, V was inverted to calculate the prewhitening matrix K.

$$cov(\hat{\beta}) = \sigma^2(Z'Z)^{-1}Z'KVK'Z(Z'Z)^{-1} \tag{S4}$$

where Z = KX, is the whitened designed matrix (Graybill (1976)). Let C be the contrast matrix of interest, so the parameter estimate for the contrast changes to $C\hat{\beta}$. Now after prewhitening, assuming unit variance $(\sigma^2 = 1)$, and from the property of Moore–Penrose inverse $(A^- = (A'A)^{-1}A')$, Equation S4 reduces to,

$$cov(C\hat{\beta}) = CZ^-KVK'(Z^-)'C' \tag{S5}$$

Now, from Equation S5, the less the variance of the parameter estimates, the more optimized the experimental design is. Hence, the following expression parameterize the efficiency of a design and is used as a measure of optimality for our simulated designs:

$$\xi = \frac{1}{trace\{cov(C\hat{\beta})\}} \tag{S6}$$

ξ is the detection power (ξ_d) if X is a convolved design matrix. ξ is the estimation efficiency (ξ_e) if X is a finite impulse response (FIR) matrix of the HRF.

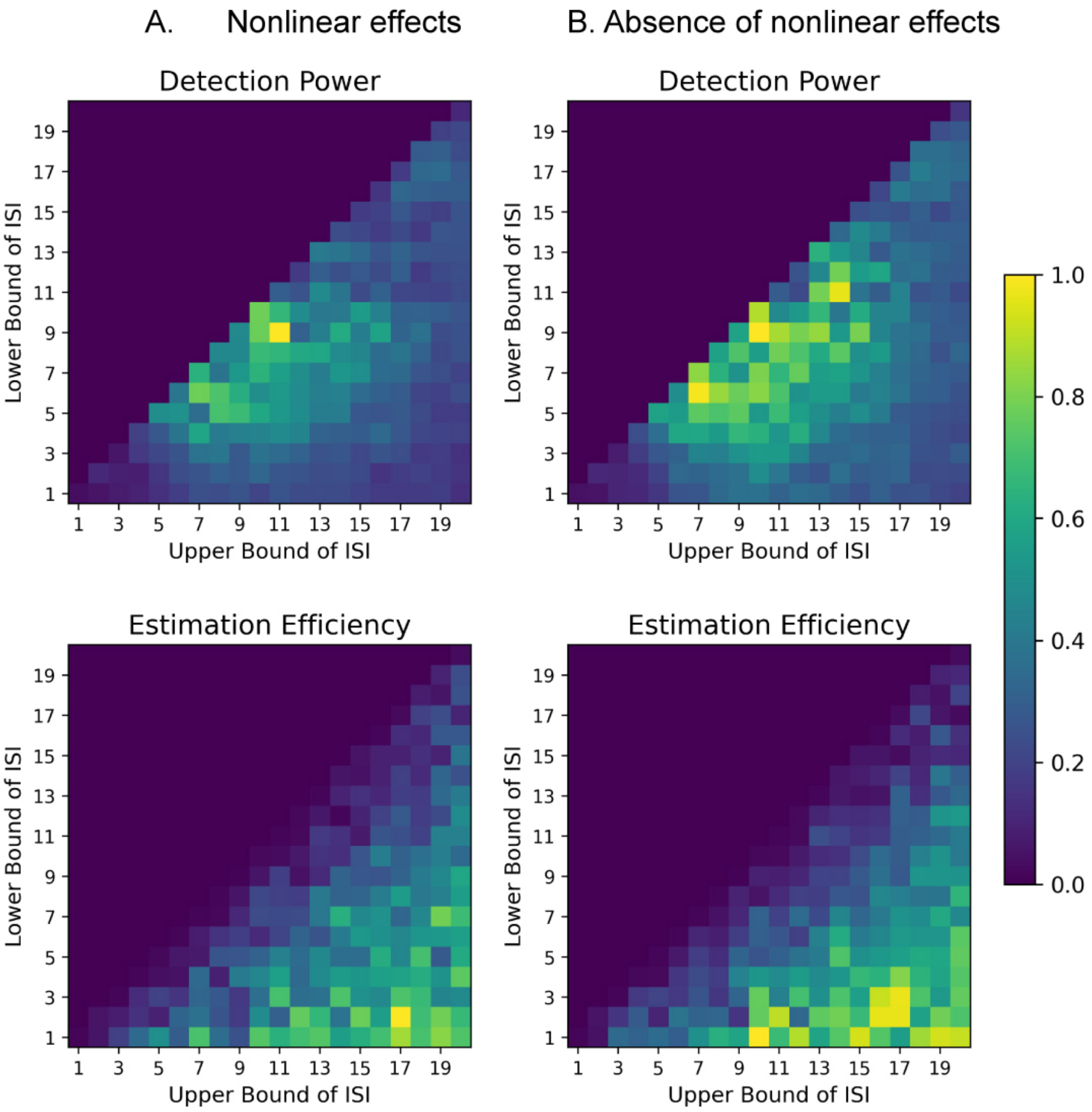

Figure S1. The effect of nonlinear interactions on the optimality measures. (A) Nonlinear interactions present **(B)** Nonlinear interactions absent

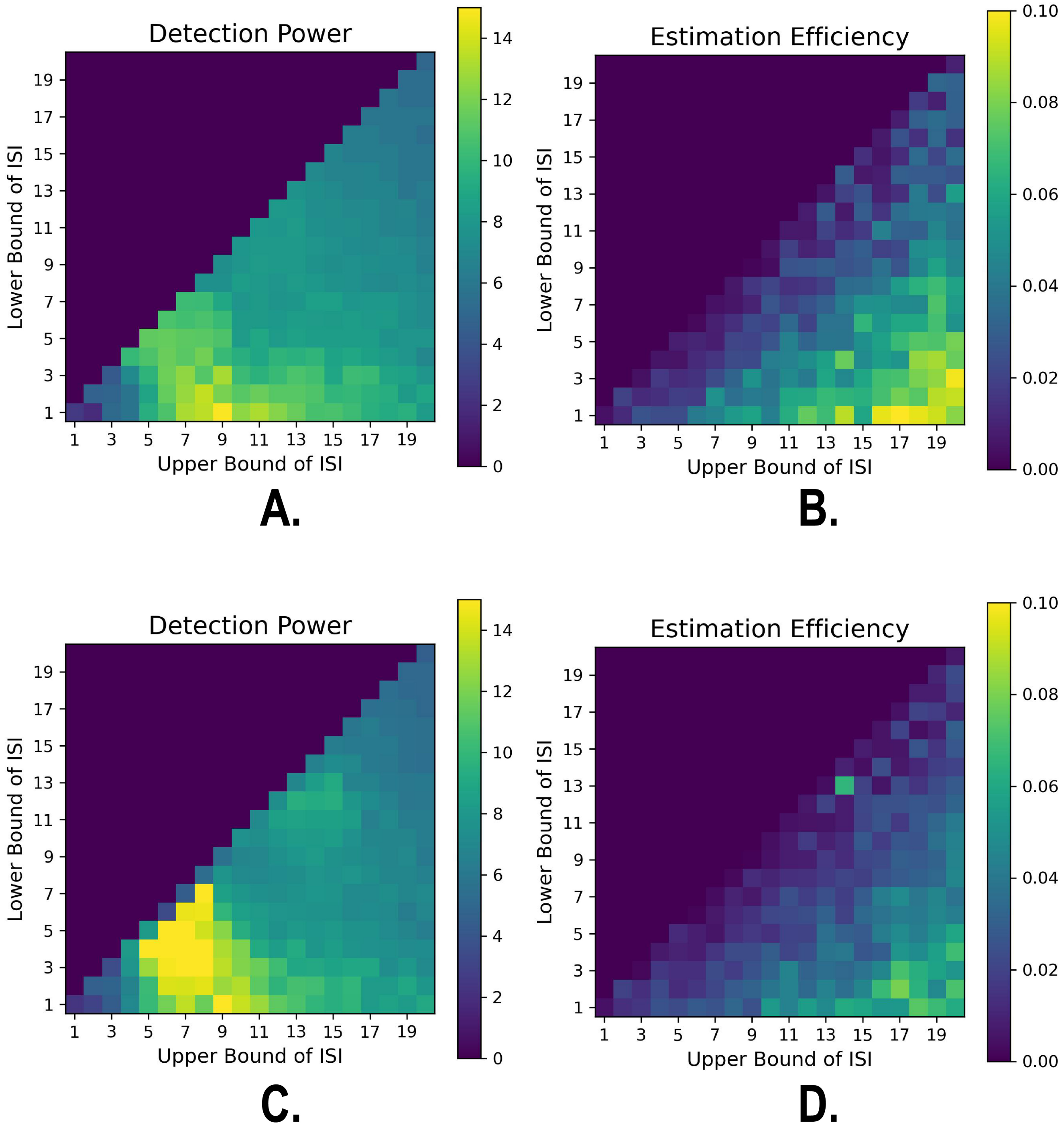

Figure S2. Optimality measures from Simulation 1 without noise. (A, B)- Optimality measures for Attention TTPs (1-4). **(C, D)-** Optimality measures for Working Memory TTPs (5-6)

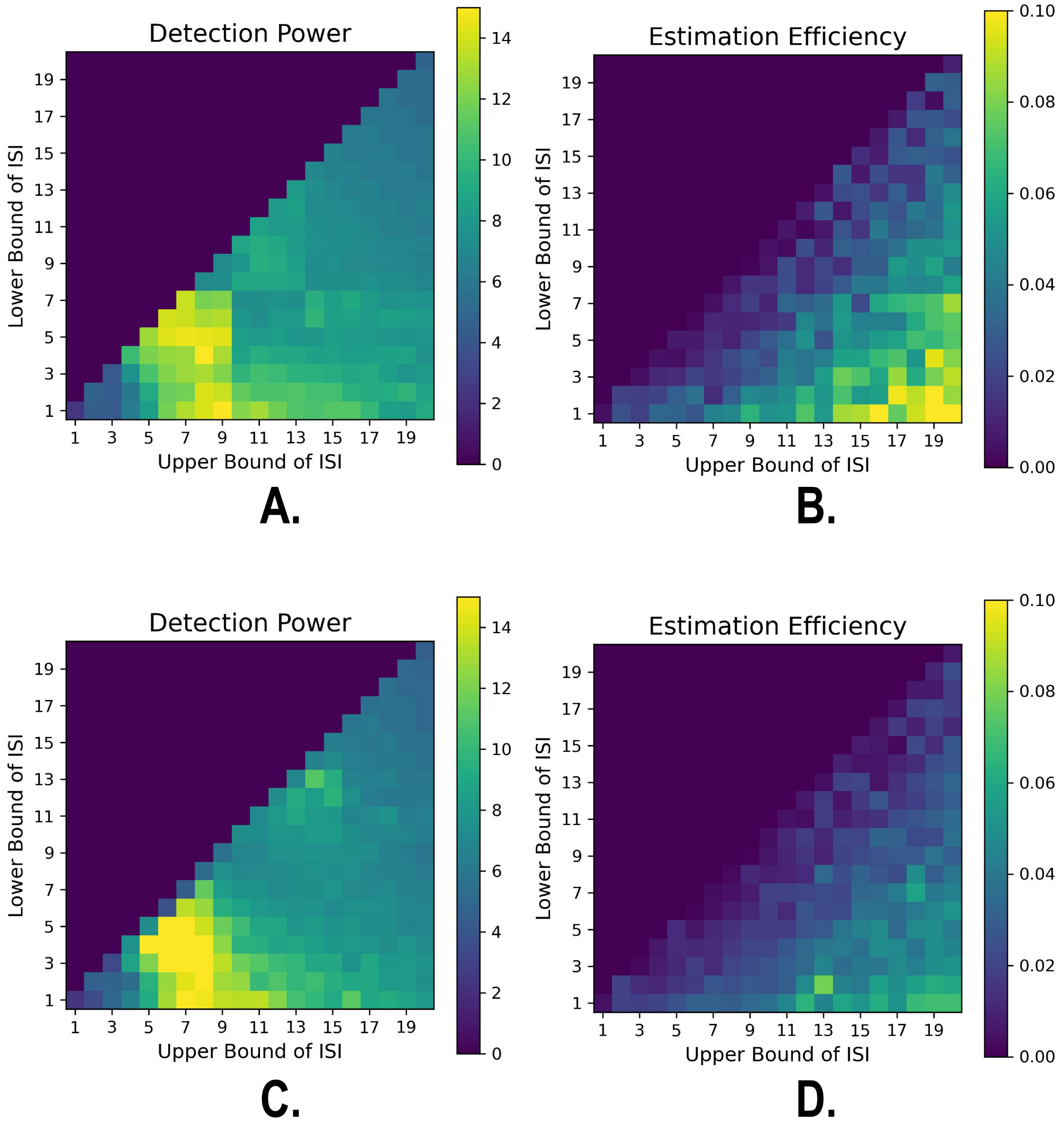

Figure S3. Optimality measures from Simulation 1 without noise, when the sub-impulses were set at *0.33* (A, B)- Optimality measures for Attention TTPs (1-4). **(C, D)-** Optimality measures for Working Memory TTPs (5-6)

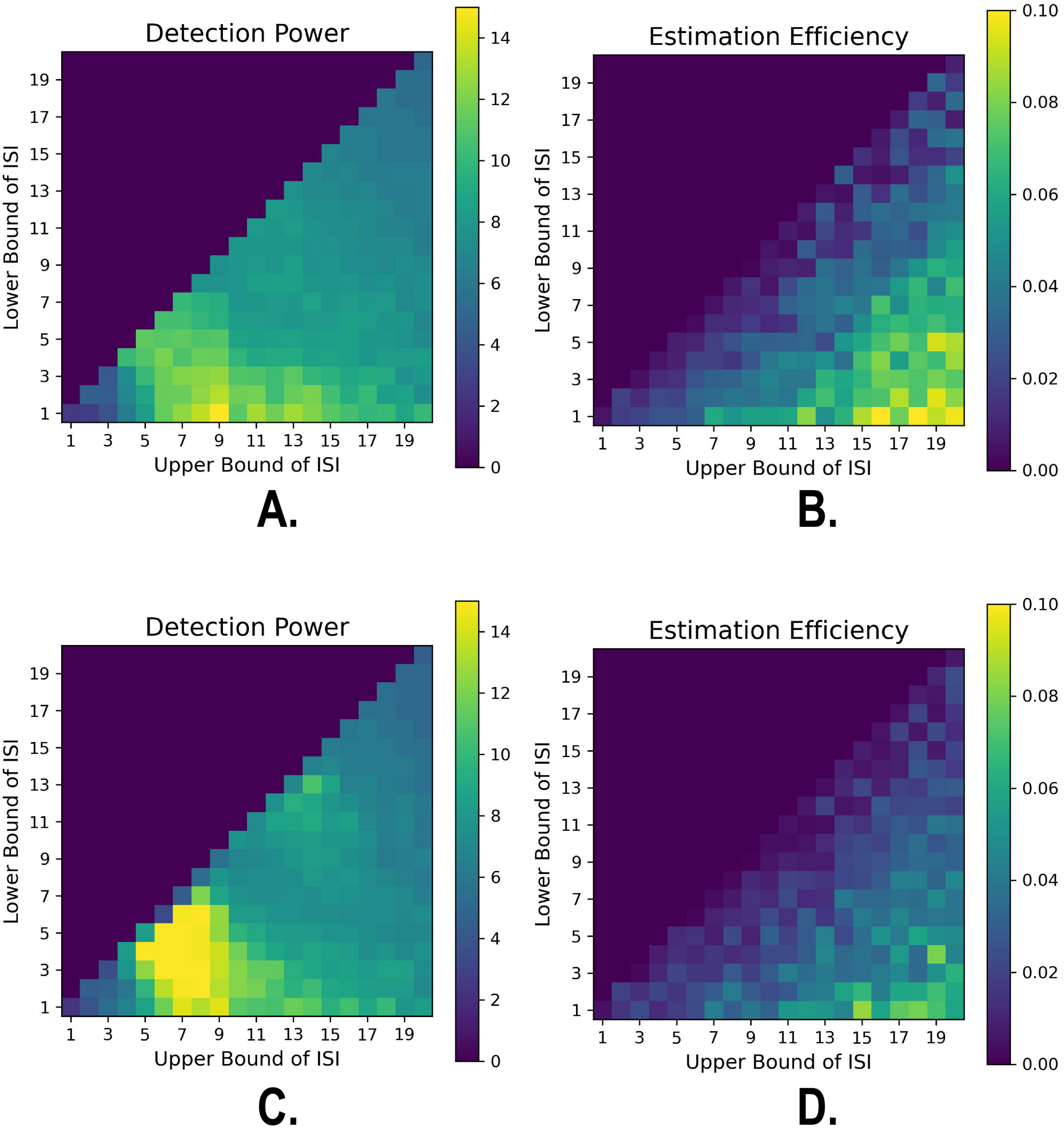

Figure S4. Optimality measures from Simulation 1 without noise, when the sub-impulses were set at *0.8* (A, B)- Optimality measures for Attention TTPs (1-4). **(C, D)-** Optimality measures for Working Memory TTPs (5-6)

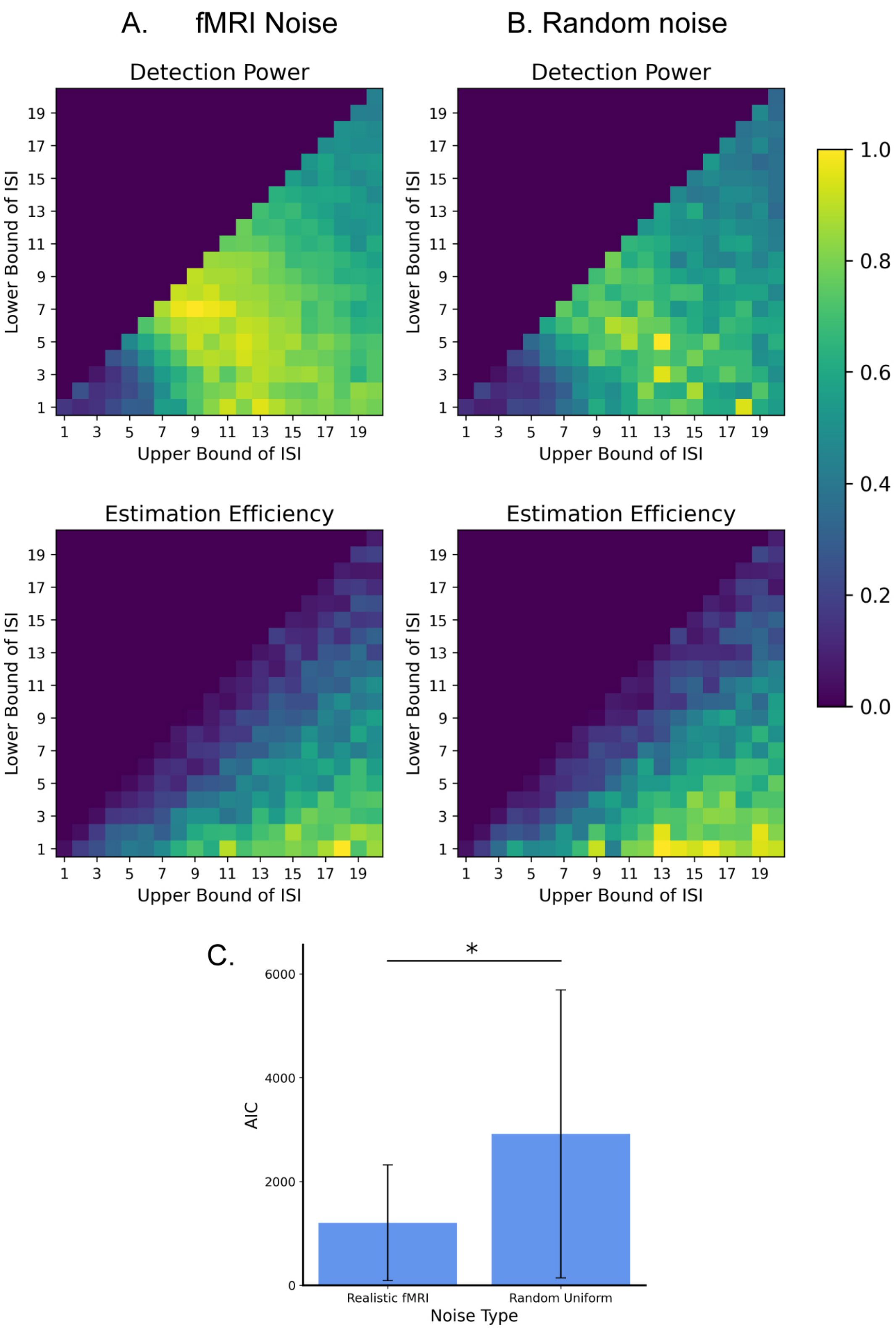

Figure S5. Optimality measures under different noise types (A) Realistic fMRI Noise **(B)** Random Uniform Noise(within ±5 SDs of the mean fMRI noise) **(C)** Akaike Information Criterion (AIC) of the GLM model fits. The estimation model fit is significantly better when using realistic fMRI noise as compared to random uniform noise (tested using a two-sided Wilcoxon Rank Sum Test $p < 0.0001$).

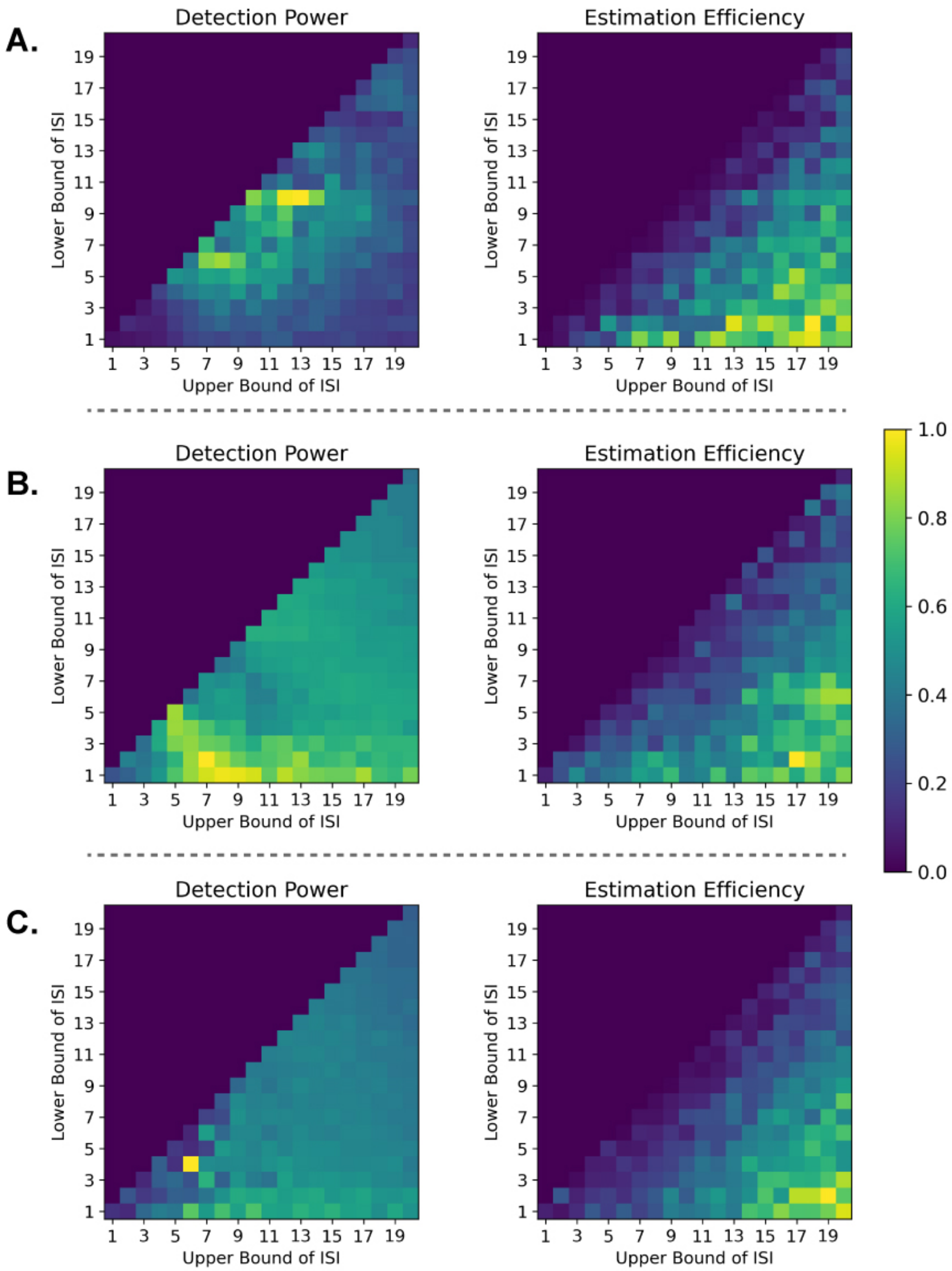

Figure S6. Optimality measures without noise in the presence or absence of TTPs (A) Optimality measures without any TTPs **(B)** Optimality measures for Attention TTPs (1-4) **(C)** Optimality measures for Working-Memory TTPs (5-6)

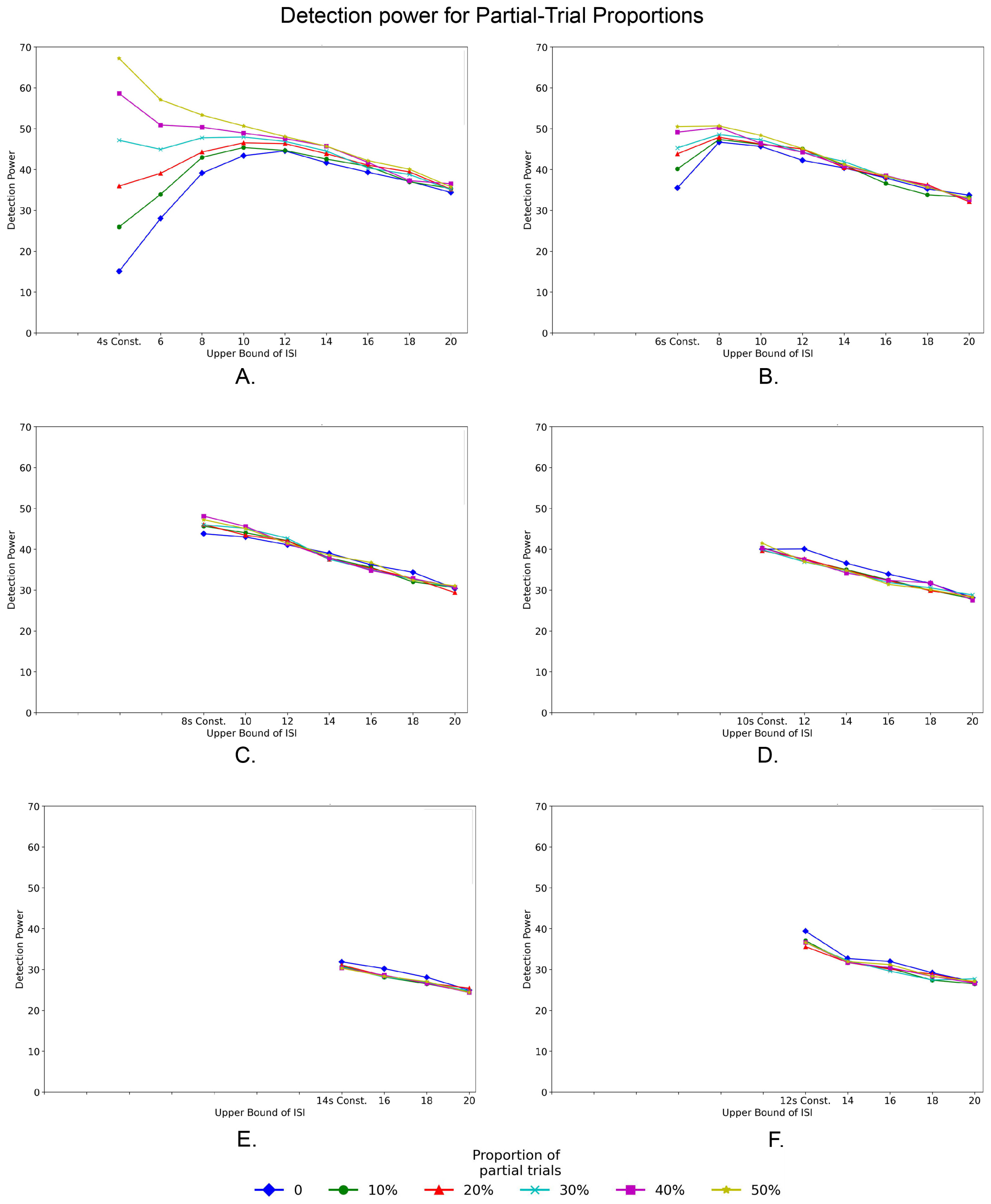

Figure S7. Detection power from Simulation 2 Optimalities are as a function of the proportion of partial trials and ISI when the lower bound of ISI was fixed at 4s (**A**), 6s (**B**), 8s (**C**), 10s (**D**), 12s (**E**), 14s (**F**)

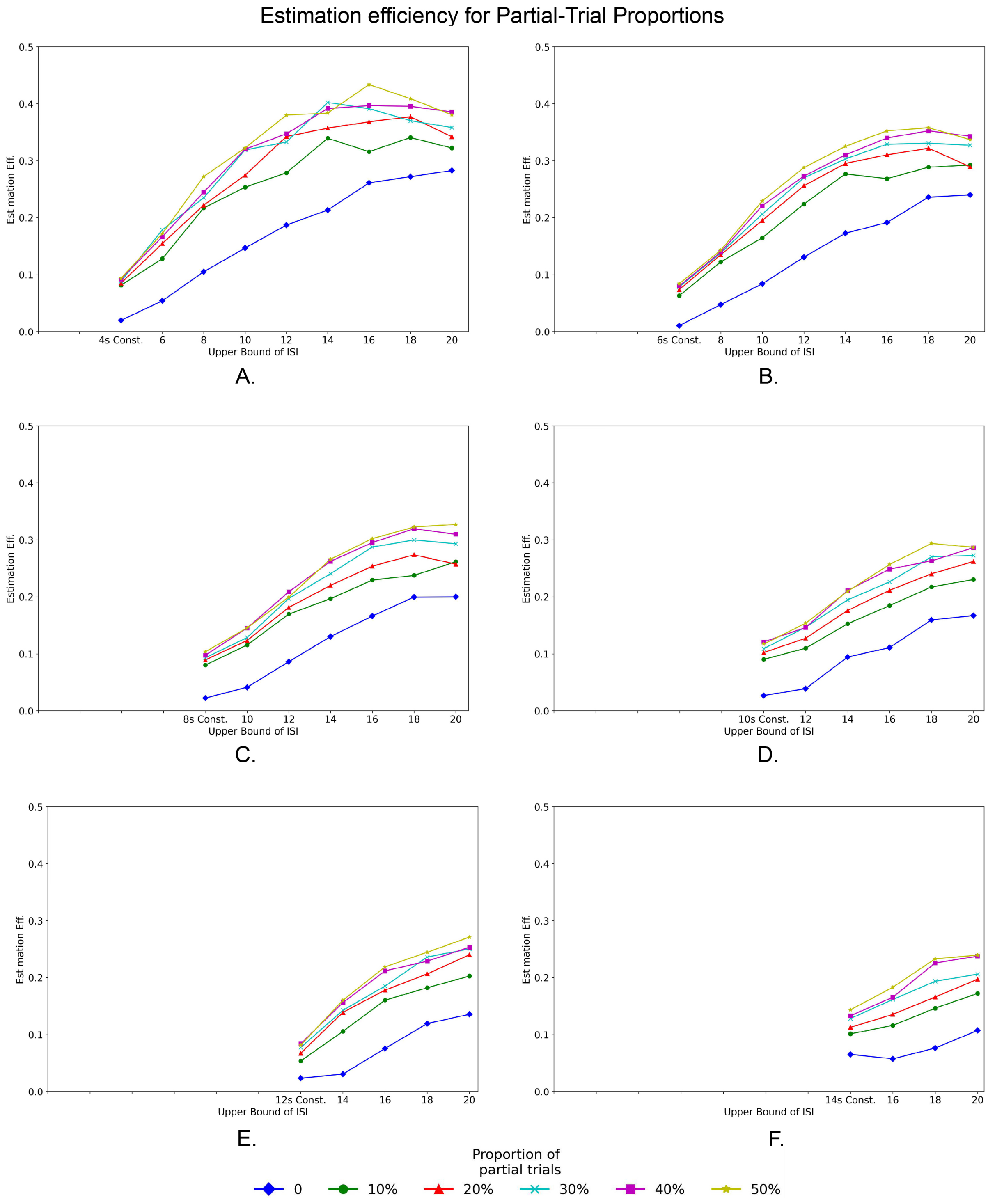

Figure S8. Estimation efficiency from Simulation 2 Optimalities are as a function of the proportion of partial trials and ISI when the lower bound of ISI was fixed at 4s (**A**), 6s (**B**), 8s (**C**), 10s (**D**), 12s (**E**), 14s (**F**)

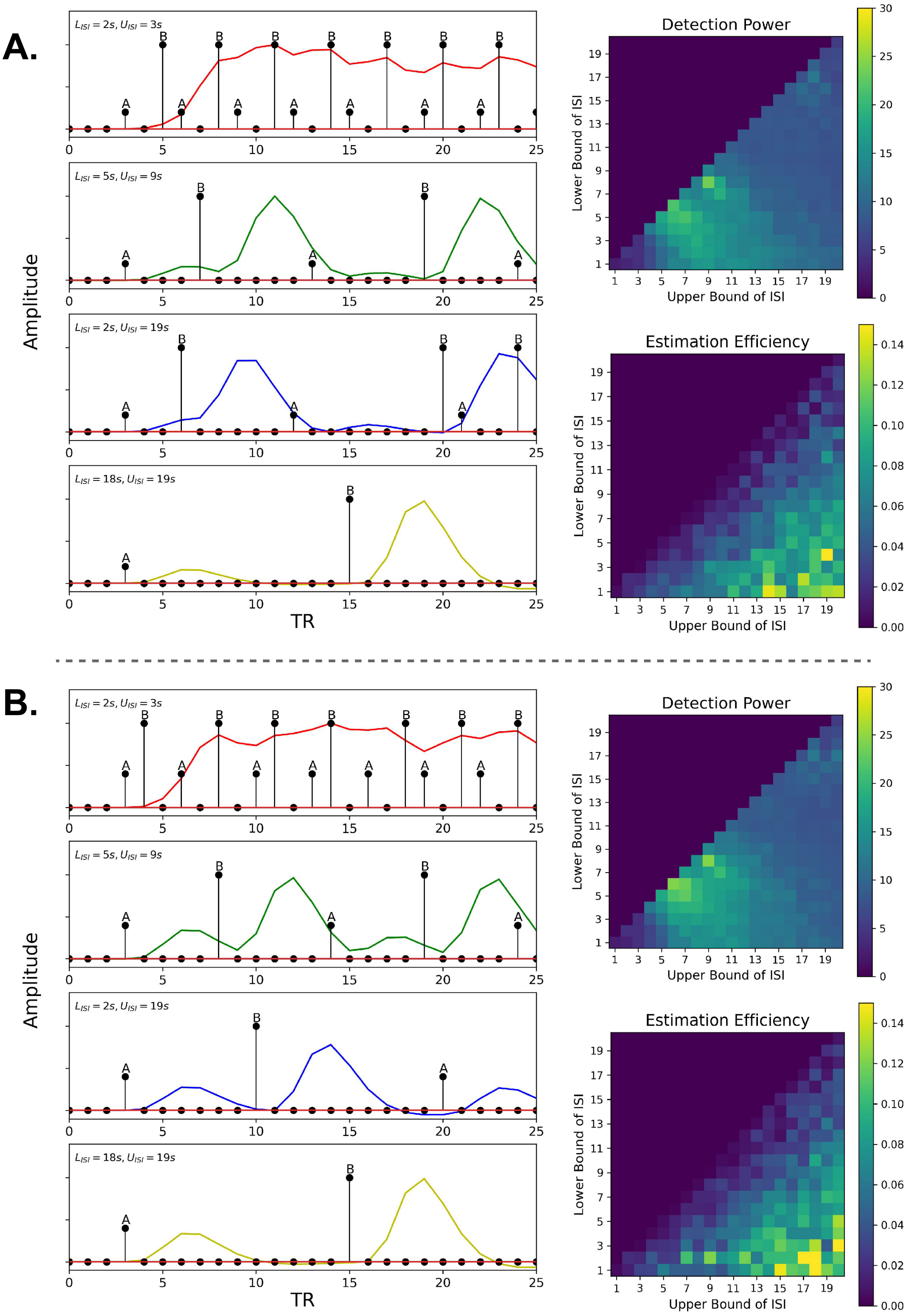

Figure S9. Time courses and optimality measures for different ratios of cue to target amplitudes. (A) Cue evoked response is *0.2* times smaller than that of target. **(B)** Cue evoked response is *0.4* times smaller than that of target.

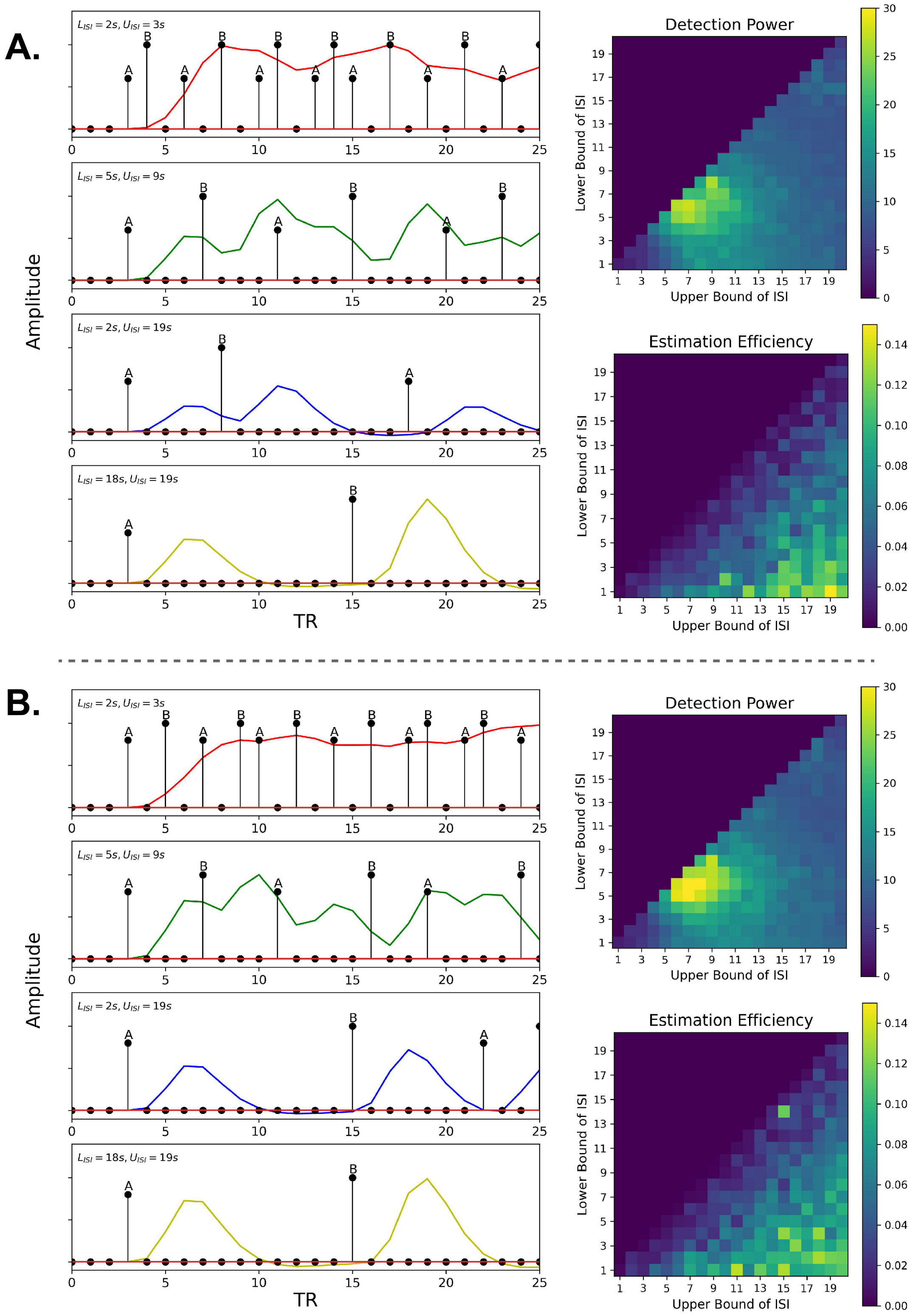

Figure S10. Time courses and optimality measures for different ratios of cue to target amplitudes. **(A)** Cue evoked response is *0.6* times smaller than that of target. **(B)** Cue evoked response is *0.8* times smaller than that of target.
